# Zinc Supplementation Reduces Common Cold Duration among Healthy Adults: A Systematic Review of Randomized Controlled Trials with Micronutrients Supplementation

**DOI:** 10.4269/ajtmh.19-0718

**Published:** 2020-04-27

**Authors:** Min Xian Wang, Shwe Sin Win, Junxiong Pang

**Affiliations:** Centre for Infectious Disease Epidemiology and Research, Saw Swee Hock School of Public Health, National University of Singapore and National University Health System, Singapore

## Abstract

The common cold had resulted in significant economic and social burden worldwide. The effect of vitamin C on preventing common cold in healthy adults has been investigated extensively, but not that of other micronutrients. Thus, we aim to assess the effects of providing micronutrients singly through oral means, on cold incidence, and/or management (in terms of cold duration and symptom severity) in healthy adults from systematically searched randomized controlled trials. From four electronic databases, 660 identified studies were screened and data were extracted from 20 studies (zinc, 10; vitamin D, 8; and vitamins A and E, 2). The quality of selected studies was assessed using the Cochrane risk of bias tool and certainty in the outcomes was assessed with the Grading of Recommendations Assessment, Development and Evaluation approach. The review found that micronutrients supplementation, except vitamin C, may not prevent cold incidence or reduce symptom severity among healthy adults. However, zinc supplementation was observed to potentially reduce cold duration by 2.25 days (when zinc is provided singly, 95% CI: −3.39, −1.12). This suggests that zinc supplementation may reduce the overall burden due to common cold among healthy adults.

## INTRODUCTION

The common cold is characterized by acute inflammation of the nose, sinuses, pharynx, and larynx mainly due to viral infection.^1^ Cold episodes are usually mild and do not progress to serious health problems, such as pneumonia or bacterial infections, or require hospitalization.^2^ However, the high morbidity of this illness, accounting for 20% of medical visits in developed countries, has caused significant socioeconomic burden through adversely affecting daily activity, productivity, and attendance from work or school.^3–6^ Globally, 6,090,503 disability-adjusted life years were lost because of upper respiratory tract infections (URTIs) in 2016.^7^

Intake of certain micronutrients enhances the immune system through strengthening epithelial barriers and cellular immunity and production of antibodies.^8^ The positive effects of micronutrient intake on the immune system suggest their potential protective role against infections.^9–14^ In the area of respiratory infections, vitamin C’s efficacy on cold prevention has been extensively studied.^15–17^ The latest meta-analysis on vitamin C’s effect on cold found that regular vitamin C supplementation in adults helped decrease cold duration by 8% (3–12%) and reduced cold severity during episodes.^14^

Vitamin D is another micronutrient that is more intensively studied in the area of cold prevention and management. However, studies presented conflicting observations^18–20^ until a recent review pooled individual participant data from primary studies to provide a more definite conclusion on vitamin D’s effect on common cold.^21^ The review indicated that vitamin D did not prevent colds in adults, regardless of health status (adjusted odds ratio [OR]: 0.93; 95% CI: 0.79, 1.10; *P* = 0.41).^21^ Although the aforementioned review analyzed data at the individual participant level, its analysis was not focused on a healthy adult population, as with the other available literature which evaluated micronutrients’ efficacy on the relevant outcomes but were rarely focused on a healthy adult population.^19,22^ The healthy adult population is often overlooked compared with the diseased and/or vulnerable (children and older) populations, where micronutrient deficiency is prevalent and supplementation had been shown to help with cold prevention and/or management.^14,23,24^ Although there are no global statistics on the prevalence of micronutrient deficiency in seemingly healthy adults, a review on the prevalence of micronutrient deficiency in the Middle East found up to 51.8% women and 27% men were suffering from vitamin D deficiency, and up to 27.2% healthy women were anemic.^25^ Thus, there is a possibility that healthy adults may benefit from micronutrient supplementation, especially if they were already deficient. From the public health standpoint, it is also important to prevent and/or manage colds in the healthy adult population, to maintain their good health status and reduce public health burden. Moreover, the evidence of other micronutrients supplementation on the management and protection against colds has also been limited. Thus, there is a need to systematically assess the existing literature, specifically randomized controlled trials (RCTs), to better understand the importance and effects of all micronutrients, except vitamin C, in fending off and managing colds among healthy adults, other than in diseased and/or vulnerable populations. This systematic review aims to assess whether 1) regular oral supplementation of a micronutrient singly prevents cold incidence or 2) oral provision of a micronutrient singly when infected with cold decreases its duration or severity among healthy adults aged between 18 and 65 years. In addition, we aim to assess the certainty of the evidence collected for each outcome of interest, when possible, using the Grading of Recommendations Assessment, Development and Evaluation (GRADE) framework.

## MATERIALS AND METHODS

### Literature search and study selection.

The systematic search methods were carried out in accordance with the relevant guidelines and regulations in the preferred reporting items for systematic reviews and meta-analyses (PRISMA) guidelines in this systematic review. For each micronutrient of interest, a literature search identifying relevant studies published in PubMed, Cochrane Library, Embase, and Scopus was carried out within the month of August 2018 using specific search terms defined with the population, intervention, comparator, outcome, study design criteria (Table 1). Specific search strategies applied to each database are provided in Supplemental Tables S1 and S2. No restrictions were placed on year and language, and reference lists of relevant reviews were also hand-searched to identify additional studies.

**Table 1:** Description of PICOS criteria for a systematic review assessing the effects of micronutrients supplementation or administration on the prevention and management of common cold and pneumonia

Variable	Description
Population	Prevention: healthy individuals aged between 18 and 65 years
	Management: individuals aged between 18 and 65 years, infected with common cold or pneumonia
	Oral administration or supplementation of a singular micronutrient.
Intervention	Micronutrients of interest: minerals (copper, iron, magnesium, selenium, and zinc) and vitamins (vitamins A, B, D, E, and K)
Comparator	Control groups without any micronutrient administration or supplementation
Outcome	Incidence, duration, or severity of common cold (primary outcome) or community-acquired pneumonia (secondary outcome)
Study design	Randomized controlled trials
Research question	Does 1) regular oral supplementation of a micronutrient singly prevent cold incidence or 2) oral provision of a micronutrient singly when infected decreases cold duration or severity among healthy individuals aged between 18 and 65 years?

PICOS = population, intervention, comparator, outcome, study design.

For each micronutrient, duplicates were removed by EndNote and manually before identified studies were screened for relevance using title and abstract. Subsequently, full texts of potential studies were screened for inclusion into the review. Studies included were 1) RCTs published in English, 2) those that involved healthy subjects (without chronic conditions/comorbidities/nonhospitalized/non–intensive care unit patients) with a mean age between 18 and 65 years who were not infected with a naturally acquired cold or community-acquired pneumonia before supplementation trial, 3) those that compared a single micronutrient that was supplemented or administered singly via oral methods with a placebo or no intervention, and 4) those that reported the incidence, duration, or severity of colds (as a primary outcome) or community-acquired pneumonia (as a secondary outcome) as outcomes of interest.

The micronutrients of interest in this review include minerals (copper, iron, magnesium, selenium, and zinc) and vitamins (vitamins A, B, D, E, and K). Studies using a combination of > 1 micronutrient as an intervention or administered a micronutrient via non-oral methods were excluded because it does not address the research question in this review. Although the common cold usually refers to URTI only, this review defines a cold episode as any URTIs, acute respiratory infections, and common cold episodes, regardless whether the illness was clinically diagnosed, laboratory-confirmed, or self-reported. Self-reported colds are defined by the presence of at least two of the symptoms in a day, which are not attributable to allergy. The symptoms include headache, fever, muscle pain, sneezing, nasal drainage, nasal obstruction, sore throat, scratchy throat, cough, hoarseness, malaise, productive sputum, or change in sputum color and quantity, nausea, and chest congestion.

### Data extraction and risk assessment.

Key characteristics and relevant outcome information were extracted from selected studies using a standardized data abstraction sheet and are summarized in Table 2. The corresponding authors of included studies were contacted for information or clarification when required. When available, the number of cold episodes, mean duration, and severity of cold episodes were evaluated as the main outcomes of this review. For studies reporting both data for self-reported colds and clinically diagnosed or laboratory-confirmed colds, only data for clinically diagnosed or laboratory-confirmed colds were recorded. For dichotomous outcomes, the number of cold outcomes and cold episodes in the intervention and placebo groups were collected. For continuous outcomes, means and SDs in duration and severity of common cold episodes in the intervention and placebo groups were collected. When these data were not reported or available after contacting authors, methods outlined in the Cochrane handbook to calculate SDs from CIs and *P*-values were used. When studies reported only median and interquartile range (IQR), the median was used to reflect the mean and the IQR was divided by 1.35 to obtain the SD.

**Table 2:** Study characteristics

Study (author, year [Ref.])	Country	Population	% Men	Mean age (SD) (years)	Intervention duration	Micronutrient form	Dosage (per capsule/tablet/lozenge); dosing schedule	Outcomes reported
Vitamins A and E (two studies)								
Hemila^27^	Finland	19,791	100	I: 57.2 C: 57.1	6.1 years	dL-α-tocopherol acetate	50 mg; NR	Self-reported cold incidence
				I: 57.3 C: 57		Water-soluble β-carotene beadlets	20 mg, NR	
Hemila^28^	Finland	26,148	100	I: 57.2 C: 57.1	6.1 years	dL-α-tocopherol acetate	50 mg; NR	Clinical pneumonia incidence
				I: 57.3 C: 57		Water-soluble β-carotene beadlets	20 mg, NR	
Vitamin D (eight studies)								
De Gruijl^29^	The Netherlands	70	8.6	I: 21.9 (2.3) C: 21.5 (2.1)	2 months	Vitamin D3	1 000 IU; 1 capsule daily	Self-reported cold incidence
Goodall^30^	Canada	600	36.3	I, C: 19	2 months	Vitamin D3	10,000 IU; 1 capsule weekly	Self-reported and laboratory-confirmed cold incidence
								Cold duration
								Subjective symptom severity per cold episode
Laaksi^31^	Finland	164	100	I, C: 18–28*	6 months	Vitamin D3	400 IU; 1 capsule daily	Self-reported cold incidence
Li-Ng^32^	United States	148	20.3	I: 59.3 (13) C: 58.1 (13.4)	3 months	Vitamin D3	2 000 IU; 1 capsule daily	Self-reported and laboratory-confirmed URTI incidence
								URTI duration
								Subjective symptom severity per URTI episode
Murdoch^33^	New Zealand	322	25.2	I: 47 (10) C: 48 (10)	18 months	Vitamin D3	200,000 IU (first 2 months) and 100,000 IU (subsequent months); 1 capsule monthly	Self-reported URTI incidence
								URTI duration
								Subjective symptom severity per URTI episode
Rees^34^	United States	759	57.7	I: 60.7 (6.7) C: 60.5 (6.4)	18 months	Vitamin D3	1 000 IU; 1 capsule daily	Self-reported cold incidence
								Cold duration
Shimizu^26^	Japan	215	30.7	I: 52.8 (6.2) C: 52.6 (67)	5 months	Vitamin D3	400 IU; 1 capsule daily	Self-reported URTI incidence
								URTI duration
								Subjective symptom severity per day while sick
Simpson^35^	Australia	34	41.2	I: 35 (12.5) C: 30.3 (11.8)	5 months	Vitamin D3	2 000 IU; 1 capsule daily	Self-reported and clinically diagnosed ARI
								Incidence
								ARI duration
								Subjective symptom severity per ARI episode
Zinc (10 studies)								
Douglas^36^	Australia	63	42.9	I: 30 C: 35.6	Until symptom resolution (min: 3 days; max: 6 days)	Zinc acetate	10 mg ‡; 1 lozenge every 2 hours while awake and between 6 and 8 lozenges daily	Cold duration
								Subjective symptom severity per cold episode
Eby^37^	United States	65	53.8	I: 35.6 (2.2) C: 28 (2.8)	Until symptom resolution	Zinc gluconate	23 mg; 2 tablets initially and followed by 1 tablet every 2 hours while awake (≤ 12 tablets daily)	old duration
								Subjective symptom severity per cold episode
Godfrey^38^	United States	73	60.3	I: 21.2 † C: 20.1 †	Until symptom resolution	Zinc gluconate trihydrate	23.7 mg; 1 lozenge every 2 hours (≤ 8 lozenges daily)	Cold duration
								Subjective symptom severity per day while sick
Mossad^39^	United States	99	19.2	I: 37.5 (7.5) C: 37.9 (9.2)	Until symptom resolution	Zinc gluconate	13.3 mg; 1 lozenge every 2–3 hours while awake	Cold duration
								Subjective symptom severity per day while sick
Petrus^40^	United States	101	46.5	I, C: 26.5	Until symptom resolution (max: 14 days)	Zinc acetate	9 mg ‡; 1 lozenge every 1.5 hours while awake (first day) and then every 2 hours while awake (on following days with symptoms)	Cold duration
								Subjective symptom
								severity per day while sick
Prasad^41^	United States	48	37.5	I: 36.4 (11.1) C: 37.8 (10.9)	Until symptom resolution	Zinc acetate dihydrate	42.96 mg; 1 lozenge every 2–3 hours while awake	Cold duration
								Subjective symptom severity per day while sick
Prasad^42^	United States	50	32	I: 34.5 (14.06) C: 35.9 (13.4)	Until symptom resolution	Zinc acetate	13.3 mg; 1 lozenge every 2 hours while awake	Cold duration
								Subjective symptom severity per day while sick
Turner^43^	United States	279	NR	NR	Until symptom resolution (min: 3 days; max: 6 days)	Zinc acetate	5 mg; 1 lozenge every 2–3 hours while awake (≤ 6 lozenges daily)	Cold duration (median)
						Zinc acetate	11.5 mg; 1 lozenge every 2–3 hours while awake (≤ 6 lozenges daily)	
								Subjective symptom severity per day while sick
						Zinc gluconate	13.3 mg; 1 lozenge every 2–3 hours while awake (≤ 6 lozenges daily)	
Veverka^44^	United States	30	80	I: 18.5 (0.89) C: 18.6 (0.82)	Supplementation: 7 months	NR	15 mg; 1 tablet daily	Self-reported and clinically diagnosed cold incidence
Weismann^45^	Denmark	130	NR	I, C: 18–65*	Until symptom resolution (max: 100 days)	Zinc gluconate	31.3 mg; 1 lozenge every 1–1.5 hours while awake (≤ 10 lozenges daily)	Cold duration
								Subjective symptom severity per cold episode
								Subjective symptom severity per day while sick

C = control; I = intervention; Max, maximum; Min, minimum; NR = not reported.

* Age range.

† Median age.

‡ Elemental zinc content.

The risk of bias in included studies was assessed using the Cochrane risk of bias tool. The certainty of evidence for each outcome was also assessed using the GRADE approach (GRADE). The approach examines the trials included for each outcome on the grounds of 1) study design; 2) risk of bias; 3) inconsistency, indirectness, and imprecision in outcome measures; and 4) the presence of publication bias, large effect magnitude, plausible confounding, and dose-response gradient, to determine the level of confidence in the summary statistic through discussion between reviewers.

Study selection, data extraction, and risk of bias assessment were performed in duplicate by the two reviewers (M. X. W. and S. S. W.). Discrepancies were discussed and resolved by consensus at the end of each procedure with a third reviewer (J. P.).

### Statistical analysis.

To use the GRADE approach for assessing certainty in outcomes, summary statistics, heterogeneity assessment, and publication bias analysis were conducted for each outcome despite the small number of studies. For the number of cold outcomes in the intervention and placebo groups, the risk ratio (RR) with 95% CI was calculated for each study. For cold duration, mean differences in cold duration between the intervention and placebo groups were pooled using a random effects model, and the result was reported as weighted mean difference and 95 % CIs. The *I*^2^ statistic and Cochran’s Q test was used to evaluate statistical heterogeneity, where heterogeneity was characterized as minimal (< 25%), low (25–50%), moderate (50–75%), or high (> 75%) and was significant if *P*-value < 0.05. When more than two studies reported the same outcome, publication bias for the outcome was assessed with contoured funnel plots. All statistical tests were two-sided and performed using Review Manager 5.3 and STATA (version 13.0; StataCorp, College Station, TX).

## RESULTS

### Screening results.

**Figure 1.**
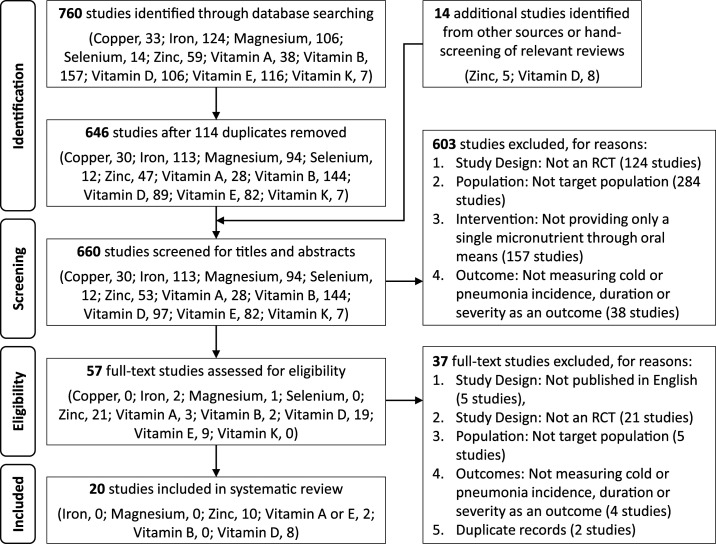
PRISMA flow diagram for study selection.

After the removal of duplicates within each micronutrient, the titles and abstracts of 660 unique studies identified through our literature search were screened, and full texts of 56 potential studies were further assessed for eligibility. As each micronutrient was screened independently, duplicate records across micronutrients were not removed. Thus, two studies were excluded at the final screening stage as the selected studies for vitamins A and E were duplicates of each other. Eventually, 20 studies were selected for inclusion into our review, where zinc (10 studies), vitamins A and E (two studies), and vitamin D (eight studies) ere each assessed for their effects on cold prevention and/or management. Other reasons for exclusion at all stages of screening are detailed in Figure 1, and no relevant studies were selected for copper, iron, magnesium, selenium, and vitamins B and K.

### Characteristics of included studies.

The key characteristics of studies included in this review are presented in Table 2. Across the 22 studies included, there were 49,189 subjects with mean age ranging from 18.5 years old to 60.7 years old, and 45,939 subjects (93.4%) were attributed to the four studies supplementing vitamin A and vitamin E, whereas 3,250 subjects (6.6%) were attributed to the 18 studies using vitamin D or zinc as an intervention. All studies used parallel designs and were conducted in non-Asian countries (Finland, the Netherlands, Canada, United States, New Zealand, Australia, and Denmark), except for one study conducted in Japan.^26^

Intervention durations, dosage, and dosing frequency varied widely between different micronutrients. For vitamins A and E, 20 mg of water-soluble β-carotene beadlets and 50 mg dl-α-tocopherol acetate was, respectively, supplemented for 6.1 years. For vitamin D, eight studies supplemented vitamin D3 between 2 months and 18 months. Vitamin D3 capsules were provided in doses ranging from 100 IU daily to 20,000 IU weekly and 100,000 IU monthly. For zinc, 10 studies provided zinc lozenges or capsules with 5 mg to 42.9 mg of zinc, mainly as zinc gluconate or zinc acetate, between 3 and 100 days or until symptom resolution for cold management, and 7 months for cold prevention.

It is interesting to note that main outcomes reported by studies using vitamins A and E as interventions were cold or pneumonia prevention, whereas studies using zinc as an intervention mainly focused on reporting cold management as an outcome and studies using vitamin D as an intervention reported both as outcomes.

### Risk of bias assessment.

The risk of bias in individual studies are presented in Table 3. Most zinc studies provided inadequate details on random sequence generation and allocation concealment methods. Across all studies, there was a high risk of other bias mainly due to underpowered studies^26,31,32,44^ or the lack of clinical or laboratory confirmation for cold resolution.^26,27,32,34,36,40,43,45^ Although these factors also exist in other studies, more details are required to assess the overall risk of other bias, such as detection and recall biases, in these studies.^29,35,45^ For the outcomes of cold incidence and duration, the risk of bias is summarized in the GRADE evidence profile (Supplemental Table S3).

**Table 3:** Risk of bias review of included studies

Type of bias	Selection		Performance		Detection	Attrition	Reporting	Other bias
Study (author, year)	Random sequence generation	Allocation concealment	Blinding: participants and research personnel	Group comparability	Blinding: outcome assessors	Incomplete outcome data	Selective reporting	
Vitamins A and E								
Hemila, 2002	Low	High	Low	Low	High	High	Unclear	Unclear
Hemila, 2004	Low	High	Low	Low	Low	High	Unclear	Unclear
Vitamin D								
De Gruijl, 2012	Low	Low	High	Low	Low	Low	Low	Unclear
Goodall, 2014	Low	Low	Low	Low	Low	Low	Low	Low
Laaksi, 2010	Low	Low	Low	Low	Low	High	Low	High
Li-Ng, 2009	Low	Low	Low	Low	Low	Low	Low	High
Murdoch, 2012	Low	Low	Low	Low	Low	Low	Low	Low
Rees, 2013	Low	Low	Low	High	Low	Low	Unclear	High
Shimizu, 2018	Low	Unclear	Unclear	Low	Low	Low	Low	High
Simpson, 2015	Low	Low	Low	Low	Low	Unclear	Low	Unclear
Zinc								
Douglas, 1987	Unclear	Low	Low	Low	Low	High	Unclear	High
Eby, 1984	Unclear	Unclear	Unclear	Unclear	Low	High	Low	High
Godfrey, 1992	Low	Low	Low	Low	Low	Low	Unclear	Unclear
Mossad, 1996	Low	Low	Unclear	Unclear	Low	Low	Low	High
Petrus, 1998	Unclear	Unclear	Unclear	Unclear	Low	Low	Unclear	High
Prasad, 2000	Unclear	Unclear	Low	High	Low	Low	Low	Low
Prasad, 2008	Unclear	Unclear	Low	Unclear	Low	Unclear	Low	Low
Turner, 2000	Unclear	Unclear	Low	Low	Low	Unclear	Low	High
Veverka, 2000	High	High	Low	Low	Low	Unclear	Unclear	High
Weismann, 1990	Unclear	Unclear	Unclear	Low	Unclear	High	High	Unclear

### Effects on cold prevention.

#### All micronutrients:

**Figure 2.**
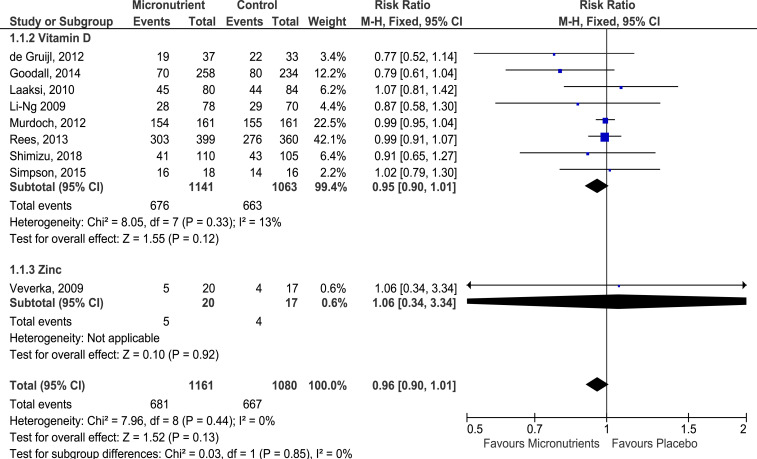
Forest plot of comparison of micronutrients versus placebo on acquiring colds. A study was excluded from the analysis as required data were not reported.^25^ Grading of Recommendations Assessment, Development and Evaluation quality of evidence: all micronutrients and vitamin D—very low. This figure appears in color at www.ajtmh.org.

Overall, 10 studies (1 of vitamins A and E, 8 of vitamin D, and 1 of zinc) reported the effects of micronutrient supplementation on cold prevention as an outcome. However, a study did not report the cold episodes occurring in the intervention and placebo groups.^27^ Thus, only data from nine studies^26,29–35,44^ were pooled to obtain an estimated summary risk (Figure 2). Overall, there were 1,348 first episodes of colds across nine studies. Compared with placebo, a 4% drop in the risk of experiencing a cold was observed when either vitamin D or zinc was supplemented (RR: 0.96), but the risk reduction was not statistically significant (95% CI: 0.90, 1.01). Across studies, heterogeneity was not significant (*I*^2^ = 0%, *P* > 0.05), but publication bias was strongly suspected as funnel asymmetry was observed in the areas with low- and mid-statistical significance in the contoured funnel plot (Supplemental Figure S1). Furthermore, serious design limitations and serious imprecision existed for studies reporting this outcome. Hence, the certainty of evidence for cold prevention through micronutrient supplementation was considered very low (Supplemental Table S3).

#### Zinc:

Only one study assessed the effects of zinc supplementation on cold prevention.^44^ The study was carried out among U.S. military cadets and concluded that orally supplementing 15 mg zinc gluconate daily for 7 months did not have any significant effect on cold prevention in an individual (RR: 1.06, 95% CI: 0.34, 3.34). However, the study did find that cold incidence frequency, reported through a weekly survey, was 11% less frequent in the zinc group than in the placebo group (zinc group: 56.7%, 135 self-reported cold episodes of 238 survey entries; placebo group: 67.9%, 163 self-reported cold episodes of 240 survey entries).

#### Vitamin D:

Supplementation of vitamin D3 in eight studies^26,29–35^ showed a nonsignificant reduction in cold incidence risk by 5% in the vitamin D group compared with the placebo group (RR: 0.95, 95% CI: 0.90, 1.01) (Figure 2). In addition, minimal but nonsignificant heterogeneity was observed across the eight studies (*I*^2^ = 13%, *P* = 0.33) and the certainty of evidence for cold prevention through vitamin D supplementation was very low, for the same reasons as the certainty of evidence for all micronutrients preventing cold (Supplemental Table S3).

There were four studies reporting cold frequency among subjects.^26,32,33,35^ In these four studies, the mean frequency of colds was higher in the placebo group than in the vitamin D group (placebo group: 2.24 colds/subject, 755 cold episodes reported among 337 subjects; vitamin D group: 2.03 colds/subject, 720 cold episodes reported among 354 subjects).

#### Vitamins A and E:

The study supplementing vitamins A and E to its subjects concluded that supplementation had no effect on cold prevention (vitamin A, RR: 1.00, 95% CI: 0.99, 1.02; vitamin E, RR: 1.00, 95% CI: 0.98, 1.01).^27^ However, the yearly cold frequency was lower when vitamin E was supplemented, relative to the placebo group (vitamin E group: 0.856 colds/year; placebo group: 0.858 colds/year). By contrast, the vitamin A–supplemented group reported a relatively higher frequency of colds than the placebo group (vitamin A group: 0.847 colds/year; placebo group: 0.844 colds/year).

### Effects on pneumonia prevention.

#### Vitamins A and E:

Only one study reported the effects of micronutrient supplementation on pneumonia prevention.^28^ The study found a 2% decrease and 3% increase in risk for pneumonia incidence when 20 mg vitamin A or 50 mg vitamin E were, respectively, supplemented for 6.1 years (median). However, none of the changes in pneumonia risk were statistically significant (vitamin A, RR: 0.98, 95% CI: 0.84, 1.13; vitamin E, RR: 1.03, 95% CI: 0.89, 1.19). Supplementation of vitamin A observed a higher frequency of pneumonia episodes in the placebo group than in the vitamin A group (placebo group: 358 cases, incidence rate: 4.7 cases/1,000 person-years; vitamin A group: 347 cases, incidence rate: 4.6 cases/1,000 person-years). However, when vitamin E was supplemented, a relatively higher pneumonia frequency was observed in the vitamin E group than in the placebo group (vitamin E group: 358 cases, incidence rate: 4.7 cases/1,000 person-years; placebo group: 347 cases, incidence rate: 4.6 cases/1,000 person-years).

### Effect on cold duration.

#### All micronutrients:

**Figure 3.**
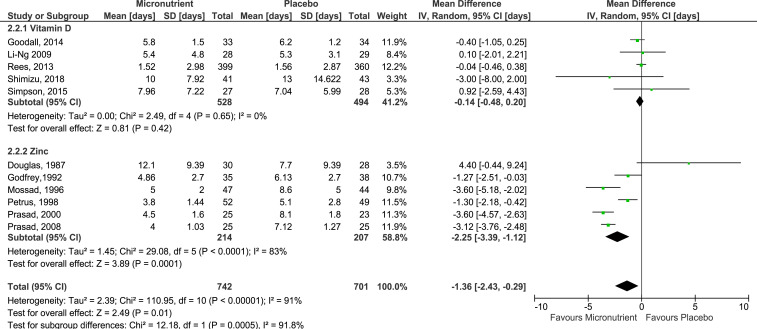
Forest plot of comparison of micronutrients versus placebo on cold duration. Four studies were excluded from the analysis as the mean number of days ill was not used as the measure for cold duration. These studies expressed the cold duration in terms of percentage of subjects symptomatic after 1 or 7 days,^35^ median number of days ill (without reporting the interquartile range),^31,41^ or survival curves.^43^ Grading of Recommendations Assessment, Development and Evaluation quality of evidence: all micronutrients, vitamin D, and zinc—low. This figure appears in color at www.ajtmh.org.

A total of 15 studies (six of vitamin D and nine of zinc) reported cold duration as an outcome, but only 11 studies could be pooled to obtain an estimated mean difference in cold duration. The pooled summary estimate of 11 studies^26,30,32,34–36,38–42^ showed a significant reduction in cold duration by 1.36 days (95% CI: −2.43, −0.29) when micronutrients, specifically vitamin D or zinc, was administered on cold infection (Figure 3). However, between-study variability was significantly high (*I*^2^ = 91%, *P* < 0.00001). As a result of high heterogeneity and serious design limitations of studies reporting this outcome, the certainty of evidence for all micronutrients on reduction of cold duration is low. Results from four studies could not be pooled as cold duration was reported in terms of percentage of subjects symptomatic after 1 or 7 days,^37^ medians without the IQR,^33,43^ or survival curves,^45^ rather than the mean number of days ill with cold.

#### Zinc:

Based on pooled results from the 6 studies,^36,38–42^ cold duration was estimated to be reduced by 2.25 days (95% CI: −3.39, −1.12) when zinc lozenges used to manage cold, compared with a placebo (Figure 3). In addition to serious inconsistency observed (*I*2 = 83%, *P* < 0.00001), there was a serious risk of bias due to design limitations of pooled studies. Thus, the GRADE certainty of evidence for zinc on this outcome is low.

In the studies where results could not be pooled, mixed results were observed. Eby et al.^37^ indicated that zinc administration reduced cold duration, after observing that 22% of subjects in the zinc group recovered within 24 hours of administering zinc gluconate lozenges, whereas none of the subjects in the placebo group recovered (*P* = 0.008). Eby et al.^37^ also observed that the estimated average cold duration was 3.9 days for the zinc group and 10.8 days for the placebo group, based on the average duration of an exponential decay curve. However, results from Turner et al.^43^ and Weismann^45^ indicated no significant differences in cold duration when zinc was administered to manage cold. Turner et al.^43^ did not observe significant differences in median cold durations between the zinc and placebo groups, regardless of zinc formulations used (median cold duration for zinc gluconate: 6.0 days, 5 mg zinc acetate: 6.0 days, 11.5 mg zinc acetate: 5.5 days, and placebo: 5.5 days). Likewise, Weismann et al.^45^ reported no significant difference in survival curves between the zinc and placebo groups in the first 5 days of illness, but observed a relatively lower probability of having the illness in the placebo group from the sixth day onwards.

#### Vitamin D:

The pooled mean difference using results from five studies^26,30,32,34,35^ suggested an estimated decrease in cold duration by 0.14 days. However, the reduction in cold duration did not reach statistical significance (95% CI: −0.48, 0.20), and heterogeneity was not significant between pooled studies (*I*^2^ = 0%, *P* > 0.05) (Figure 3). However, imprecision and serious risk of bias were detected in pooled studies. Thus, the GRADE certainty of evidence for vitamin D administration for reduction of cold duration is very low.

The study by Murdoch et al.,^33^ which was not pooled with the other studies as the IQR was not provided, reported no significant difference in median cold duration between the vitamin D and placebo group (12 days in both groups; RR: 0.96, 95% CI: 0.73, 1.25, *P* = 0.76).

### Effect on cold severity.

#### All micronutrients:

Effects of micronutrient administration on managing cold severity are summarized in Table 4. A total of 14 studies (five of vitamin D and nine of zinc) measured and reported cold severity as an outcome.^26,30,32,33,35–43,45^ A variety of severity scales, ranging from visual analogue scales to point scales with varying point systems, were used to measure the severity of cold symptoms. Symptoms that were commonly assessed include sneezing, nasal drainage, nasal congestion, headache, sore throat, scratchy throat, cough, hoarseness, muscle ache, and fever. Myalgia, chest congestion, and head congestion were occasionally assessed.^26,30,33,40^ A few studies did not specifically specify the symptoms evaluated when assessing cold severity.^32,35,36,45^ Across the 14 studies, severity scores were summed differently but were mostly presented either as the mean severity score per cold episode or as the mean severity score per day while ill, except in one study where it was unclear that the presented mean severity score was that for a cold episode or a day while ill.^32^

**Table 4:** Key findings on managing cold severity with micronutrients`

Study (author, year)	Severity scale used	Key findings*
Vitamin D		
Goodall, 2014	WURSS-21†	Mean severity score per episode is 48.2 ± 27.92 points higher in the vitamin D group than in the placebo group (*P* = 0.09).
Li-Ng, 2009	5-point scale (1 = healthy, 5 = “very ill” )	Mean severity score is 0.2 ± 0.24 points lower in the vitamin D group than in the placebo group (*P* = 0.4).
Murdoch, 2012	WURSS-24‡ and WURSS-21	Mean severity score per episode is lower in vitamin D the group (162 ± 1,142.2) than in the placebo group (167.5 ± 147.4) (*P* = 0.48).
Shimizu, 2018	WURSS-21	Mean severity score per day while ill is 4.5 ± 3.13 points lower in the vitamin D group than in the placebo group (*P* = 0.154).
Simpson, 2016	6-point scale (0–5, where 0 is no presence of that symptom and 5 is most severe)	Mean severity score per episode is 2.87 ± 11.38 points lower in the vitamin D group than in the placebo group (*P* = 0.4).
Zinc		
Douglas, 1987	4-point scale§	Mean severity score per episode is 1.9 ± 2.80 points higher in the zinc group than in the placebo group.
Eby, 1984	4-point scale§‖	Average total severity scores dropped significantly faster in the zinc group than in the placebo group (half-lives of exponential decay curves 1.9 ± 0.3 for the zinc group vs. 4.5 ± 1.0 for the placebo group). However, initial severity was significantly lower in the zinc group.
		Frequency and severity score was consistently lower for all symptoms in the zinc group than in the placebo group, after 7 days of zinc administration.
Godfrey, 1992	4-point scale§‖	By day 7 of zinc administration:
		Symptoms left: three mild symptoms (symptom severity score of 1 for each symptom) in one subject in the zinc group, compared with an average of 2.4 moderate symptoms (average severity score of 2.6) in eight subjects in the placebo group.
		Nasal drainage incidence: 5% in the zinc group vs. 33% in the placebo group.
		Nasal congestion: 0% in the zinc group vs. 31% in the placebo group.
Mossad, 1996	4-point scale§‖	Zinc group had significantly fewer days with any symptoms than the placebo group.
Petrus, 1998	4-point scale§‖¶	Mean severity score per day while ill is 0.09 ± 0.06 points lower in the vitamin D group than in the placebo group.
Prasad, 2000	4-point scale§‖	Severity scores were significantly different between the zinc and placebo groups after 10 days of zinc administration (*P* = 0.0002).
		Average severity scores decreased from 8.32 (baseline) to 3.45 (day 4) in the zinc group and from 7.78 (baseline) to 5.61 (day 4) in the placebo group.
Prasad, 2008	4-point scale§‖	Severity scores were significantly different between the zinc and placebo groups after 12 days of zinc administration (*P* = 0.0002).
		Average severity scores decreased from 10.8 (baseline) to 2.7 (day 4) in the zinc group and from 8.9 (baseline) to 5.4 (day 4) in the placebo group.
Turner, 2000	4-point scale§‖	No significant differences between zinc and placebo groups for the total symptom score or severity of any individual symptoms over the first 3 days of zinc administration or on any of the individual days, regardless of zinc formulations provided.
Weismann, 1990	Visual analogue scale (11 cm horizontal line) for overall condition	No significant difference in severity in both groups, even on days with largest severity difference between the zinc and placebo groups.
		No significant differences in cold severity compared with prior episodes, in both the zinc and placebo groups (*P* = 0.64)

* Scores are presented as mean score ± SEM.

† Wisconsin Upper Respiratory Symptom Survey-21 (WURSS-21) consists of 21 questions rated on a 0–7 Likert scale. Symptoms rated for physical severity include runny nose, plugged nose, sneezing, sore throat, scratchy throat, cough, hoarseness, head congestion, chest congestion, and feeling tired.

‡ Wisconsin Upper Respiratory Symptom Survey-24 (WURSS-24) consists of 24 questions rated on a 0.7 Likert scale. Symptoms rated for physical severity include headache, body ache, and fever and the other symptoms rated in WURSS-21.

§ 4-point scale: 0 = none; 1 = mild; 2 = moderate; and 3 = severe.

‖ Symptoms include sneezing, nasal drainage, nasal congestion, headache, sore throat, scratchy throat, cough, hoarseness, muscle ache, and fever.

¶ Additional symptom: myalgia.

#### Zinc:

All studies which provided zinc lozenges or tablets to manage cold severity assessed cold severity with a 4-point scale, with the exception of one study which used a visual analogue scale.^45^ Seven studies presented cold severity as the mean score per day while ill,^38–43,45^ whereas the remaining two studies presented severity as the mean score per episode.^36,37^ Compared with the placebo group, cold severity was mostly lower in the zinc group, except for three studies which either reported more severe symptoms in the zinc group^36^ or no significant difference in cold severity between the zinc and placebo groups^43,45^ (Table 3).

#### Vitamin D:

Three of the five studies which reported subjective symptom severity used the Wisconsin Upper Respiratory Symptom Survey-21 (WURSS-21)^26^,^30^,^33^ to measure cold severity, whereas the remaining two studies used 5- or 6-point systems for this purpose.^32,35^ Mean severity score was generally lower in the vitamin D group than in the placebo, regardless of whether the severity score was that for a single cold episode or single day while ill. Only one study reported a mean severity score that is higher by 48.2 ± 27.92 points in the vitamin D group, than in the placebo group.^30^ However, the statistical significance of the difference in mean severity scores between the vitamin D group and placebo groups were nonsignificant in all studies.

## DISCUSSION

Overall, micronutrients show promise in shortening cold duration, but may not be as effective in preventing colds in a healthy adult population. The efficacy of micronutrients in managing cold severity remains inconclusive, and further analysis with standardized scales and measures are required because current evidence present differential effects. Although effort was made to identify all possible micronutrients supplementation and their potential effects on prevention and management of cold, no relevant studies on the effects of copper, iron, magnesium, selenium, and vitamins B and K were identified. This result, and the low number of selected studies in our review, highlights the paucity of studies focused on micronutrient supplementation for purposes of cold prevention and/or management among the healthy adult population. The lack of studies also stresses on the need for more research focusing on these micronutrients to prevent and/or manage colds in a healthy adult population, instead of the diseased, children, or elderly patients. Thus, it is important to emphasize that the aforementioned effects on cold and pneumonia prevention only represents that of zinc and vitamins A, E, and D, whereas those on cold management only represents that of zinc and vitamin D.

### Prevention of cold and pneumonia.

Although nonsignificant reductions in cold incidence were consistently observed across all micronutrients, the effects mainly reflected those of vitamin D as most data for Figure 2 came from studies providing vitamin D (99.4%) for cold prevention purposes. It was also observed that zinc or vitamin D supplementation reduced the recurrence of colds in an individual, as seen from the relatively lower cold frequencies in the micronutrients groups, compared with the placebo groups. The studies included in Figure 2 generally had a low risk of biases in the prespecified bias domains. However, the risk of other biases was high or unclear in all but two studies^30,33^ because of suspected detection and recall biases.

#### Vitamin D

The nonsignificant reduction in cold incidence risk observed in this review (RR: 0.95, 95% CI: 0.90, 1.01) agrees with those from recent meta-analyses, although the meta-analyses used data from subjects across all ages and health statuses (OR: 0.93, 95% CI: 0.79, 1.10^21^; RR: 0.94, 95% CI: 0.88, 1.00^19^). Although factors potentially affecting cold incidence risk were not explored in this review, existing meta-analyses gave contrasting evidence about the effects of age on cold incidence probability (*P*_interaction_ = 0.05,^21^ *P*_interaction_ = 0.84^20^) and consistently suggested that differing health status may not affect cold incidence among adult subjects (*P*_interaction_ = 0.38,^21^ *P*_interaction_ = 0.24^20^). Instead, the subjects’ vitamin D status (*P*_interaction_ = 0.01 ^21^) and dosing regimen (*P*_interaction_ = 0.05,^21^ *P*_interaction_ = 0.01^20^) were likely risk factors affecting cold incidence likelihood. Significant reductions in cold incidence odds were observed when supplemented subjects were vitamin D deficient (OR: 0.58, 95% CI: 0.40, 0.82^21^) or when daily doses were provided (OR: 0.81, 95% CI: 0.72, 0.91^21^; OR: 0.51, 95% CI: 0.39, 0.67^20^), compared with when subjects were not deficient (OR: 0.81, 95% CI: 0.71, 1.04^21^) or provided with bolus doses (OR: 0.97, 95% CI: 0.862, 1.10^21^; OR: 0.86, 95% CI: 0.62, 1.20^20^). Unlike the previous meta-analyses, there was low heterogeneity between studies included in the forest plot (Figure 2). This could be attributed to the fact that all but two studies used a daily dosing schedule, thus resulting in consistent effects across the analyzed studies.^30,33^

#### Zinc

In general, zinc supplementation was shown to prevent up to 53% of common cold episodes when supplemented to healthy children younger than 10 years (RR: 0.64, 95% CI: 0.47, 0.88).^46^ However, such efficacy is not observed in healthy adults, even when a higher dosage was provided (RR: 1.06, 95% CI: 0.34, 3.34).^44^ The discrepancy could be because of the relatively more robust immune systems of adults, compared with children, when both are free of chronic comorbidities.^47^ Thus, zinc supplementation in children helps to develop and regulate their immune systems, hence effectively preventing cold episodes in this population. Similar to vitamin D, the efficacy of zinc supplementation on cold prevention could also be influenced by an individual’s zinc status.^18^ Zinc deficiency has been associated with a suppressed immune system, and the protective effects of zinc against colds have been evidenced in autoimmune subjects of all ages, of which 70% were zinc deficient.^48^ Thus, the lack of effect observed in Veverka et al.^44^ could be due to adequate zinc levels in the healthy adult subjects used in the study. Nonetheless, it remains unclear whether zinc levels in the seemingly healthy subjects recruited in the study by Veverka et al.^44^ were truly sufficient because of the lower population-specific zinc deficiency cutoff levels. Although Veverka et al.^44^ measured subjects’ zinc levels, zinc deficiency cutoff levels in the study population was not assessed and reported according to guidelines released jointly by the WHO, the United Nations Children’s Fund, the International Atomic Energy Agency, and the International Zinc Nutrition Consultative Group. According to the guidelines released, the lower cutoff level for zinc deficiency differs according to the age, gender, and time of the day the reading was taken for each population, and the cutoff value that can be applied universally to all adult humans does not exist.^49^ Thus, the possibility that zinc supplementation has no effect on cold effect in healthy adults (that are zinc deficient) still remains.

#### Vitamins A and E

The studies supplementing vitamins A and E were not shown to prevent the incidence of cold or pneumonia in this review.^27,28^ The authors of the studies demonstrated that the effects of supplementation with vitamins A or E on cold prevention is dependent on the age group, smoking habits, and living in the city or not for healthy smoking adults aged between 50 and 65 years. Smoking habits included the number of cigarettes smoked daily (light, 5–14 cigarettes; heavy, ≥ 15 cigarettes), and the age the adult started smoking. Light smokers who started smoking early (≤ 20 years old) had increased cold incidence when supplemented with vitamin A.^50^ By contrast, the effects of vitamin E supplementation generally decreased cold incidence in heavy smokers aged between 61 and 63 years, who are not living in the city.^51^ When vitamins A and E are provided together, their effect on cold incidence could be affected by the physical activity level at work in subjects with physically intensive jobs (defined as jobs with heavy physical work requiring much lifting or carrying heavy objects, digging, shoveling, or chopping wood) (*P*_interaction_ = 0.037).^52^ By contrast, supplementation of vitamin A singly or in combination with vitamin E in subjects who does heavy exercise for leisure, respectively, increases their cold incidence risk by 25% and 21%, compared with subjects in the placebo group.^52^ In addition, there was no significant difference in cold incidence risk for subjects across all levels of physical activity, either for work or leisure when supplementation status was disregarded.^52^

The effects of vitamin A supplementation on pneumonia incidence were also affected by the smoking habits of subjects. In heavy smokers (≥ 21 cigarettes daily, median: 30 cigarettes daily) who started smoking late (≥ 21 years old), vitamin A supplementation may increase pneumonia risk by 400%.^53^ Similarly, the effects of vitamin E supplementation on pneumonia risk were significantly modified by the smoking and exercise habits in subjects. Although both smoking and exercise habits affect the protective effect of vitamin E on pneumonia incidence, the latter seemed to have a stronger influence over the overall efficacy. Vitamin E supplementation to light smokers (5–19 cigarettes daily) who started smoking late decreased pneumonia risk by 69% when the subjects exercised leisurely. However, risk only decreased nonsignificantly in light smokers who did not exercise or heavy smokers (≥ 20 cigarettes daily) who exercised leisurely.^54^ Comparatively, vitamin E supplementation can prevent pneumonia incidence by an additional 3% in nonsmoking subjects.^55^ Collectively, these studies show that the effects of vitamin A and E on common cold or pneumonia incidence vary widely, but are mainly influenced by the smoking status of men aged between 50 and 69 years, and in some cases affected by the exercise habits and the age the subjects started smoking.

### Management of cold.

Generally, the provision of micronutrients when infected with cold are shown to decrease cold duration (weighted mean difference [WMD]: −1.36 days, 95% CI: −2.43, −0.29). However, this effect was not consistently observed across studies (*I*2 = 91%, *P* < 0.00001), especially when zinc was provided (*I*2 = 83%, *P* < 0.00001). Nevertheless, a significant reduction of up to 3.39 days was observed when zinc was provided singly during a cold (WMD: −2.25 days, 95% CI: −3.39, −1.12).

The decreased cold duration and inconsistent results among healthy adults observed in this review is similar to that reported in latest meta-analyses by Johnstone et al.^22^ and Singh et al.,^46^ which assessed the effects of zinc on cold duration and severity in adults. Although the heterogeneity among included studies were high in this review and in those two reviews (*I*^2^ = 82%, *P*-value unreported; *I*^2^ = 90%, *P* < 0.00001^46^), the extent of cold duration reduction observed in this review was more similar to that reported by Johnstone et al.^22^ (WMD: −2.63 days, 95% CI: −3.69, −1.58). Comparatively, a relatively smaller reduction was reported by Singh et al.^46^ (MD: −1.97 days, 95% CI: −3.09, −0.85). Nonetheless, two of the three studies not included in the forest plot presented differential results.^43,45^ Compared with the other studies which did not limit the daily number of lozenges consumed, or had a daily limit of 12 lozenges,^37^ these two studies had a relatively lower limit on the number of zinc lozenges consumed daily (daily limit: 6 and 10 lozenges). The amount of zinc consumed could have caused the lack of effect observed in these two studies. The maximum daily amount of zinc gluconate, respectively, consumed by subjects in Weismann et al.^45^ and Turner et al.^43^ is 313 mg and 79.8 mg, whereas that of subjects in Eby et al.^37^ is only 276 mg. However, a positive effect agreeing with the summary statistic in Figure 2 was only observed in Eby et al.^37^ It has been shown that at plasma levels above 50 µm, zinc acts as an immunosuppressant and alters normal immune function.^56^ Thus, it is plausible that the zinc dosages provided in Weismann et al.^45^ was so high that zinc levels exceeded the threshold suppressed immune responses, whereas that provided by Turner et al.^43^ was insufficient to stimulate immune responses. It was also noted that providing zinc lozenges within 24 hours after symptom onset was more effective in cold management than providing zinc lozenges after 24 hours.^57^ Godfrey et al.^38^ showed that cold duration was 1.42 days shorter in patients with symptoms for < 24 hours than those with symptom onset for < 48 hours, before both groups of patients were provided with zinc lozenges (*P* = 0.035). Another factor that could have resulted in differential effects was the different zinc formulations used, which results in different levels of ionic zinc available in vivo. In vivo, the zinc acetate formulations were less effective at generating zinc ions which had a direct antiviral effect on common cold viruses, compared with zinc gluconate formulations.^22,58^ Moreover, the antiviral properties of zinc are virus-specific, and hence, the virus involved in the cold episode also influences the therapeutic efficacy of the zinc lozenge in managing cold durations.^22,59^ Collectively, these factors could have contributed to the varied results observed in the studies included in this review.

In the studies included in this review, a variety of scales were used to measure symptom severity during a cold episode. In addition, the recorded raw severity scores were also processed differently before they were presented as a mean score per episode or per day while ill. In this review, we did not standardize the severity scales and scores because of the problems associated with standardization, such as a skewed proportion of absolute distances between response options in the various scales,^60^ and the different ways the severity scores were calculated in the different studies. Thus, we were unable to assess the certainty of findings using the GRADE approach for this outcome as a representative summary statistic cannot be estimated. Although validated instruments such as the WURSS-21 is available with a recommended approach to process the recorded scores, it is not commonly used, and there are still various ways of processing the recorded scores.^61^ Therefore, this highlights the need for a well-developed, standardized, and validated scale along with a standard approach of processing the severity scores to assess symptom severity in future studies. Nevertheless, qualitative evidence from the included studies administering vitamin D as to manage cold all indicated nonsignificant differences between groups, whereas studies administering zinc suggested differential effects. Similar differential effects were also observed in studies excluded from pooling (to generate a mean difference) in the latest Cochrane systematic review on this topic.^46^ Interestingly, all but one study^43^ conducted in the United States suggested that zinc administration helped reduce cold severity. By contrast, studies conducted in Australia and Europe indicated no significant reduction in severity, or even increased cold severity with zinc administration.^36,45^

Similar to our findings on the impact of zinc administration on cold severity, there still seems to be no consensus on this topic despite efforts to reach a conclusion. The two most recent meta-analyses which standardized and pooled severity scores across their selected studies reported contrasting findings between placebo and zinc-administered groups from pooled studies. Johnstone et al.^22^ reported significantly less severe symptoms in the zinc-administered group (standardized MD: −0.64, 95% CI: −1.05, −0.24), but Singh et al.^46^ reported no difference in severity scores between these two groups (MD: −1.06, 95% CI: −2.36, 0.23). This discrepancy is not expected as both meta-analyses used similar statistical methods to process the data from their selected studies and included the same studies in their pooling, with the exception of an additional study^40^ in Singh et al.,^46^ to generate a mean difference. Hence, the conflicting observation could possibly be attributed to that additional study included (as it has a relatively high weight (26.9%) in the overall mean difference generated) or the problems associated with standardization, as mentioned in the previous paragraph.

However, certain issues need to be addressed before making a decision on whether to provide these micronutrients supplementation as a long-term approach to effectively prevent and/or manage cold among healthy adults. The first issue is the safety of supplementing these micronutrients. Second, more standardized RCTs on the effects of micronutrients in healthy Asian populations are still needed to fully understand the collective and individual effects of micronutrients on cold prevention and management. Third, a standardized instrument and approach to measure symptom severity should be established for usage in future studies, to allow for the objective assessment of micronutrients’ effect on symptom severity.

### Limitations.

The huge limitation of this review is the low external validity of the results, in terms of the type of micronutrients studied, the form of micronutrients used in the trials, and the limited populations involved in the trials. Because of the lack of studies investigating the effects of providing micronutrients singly to prevent or manage cold in a healthy adult population, there was a low number of studies included in this review. This is especially so for copper, iron, magnesium, selenium, and vitamins B and K, whereby no studies were identified to be investigating the outcomes of interest with these micronutrients. Thus, the findings of this review only extend to zinc and vitamins A, D, and E for their corresponding outcomes. External validity was also limited to the effects of single micronutrients on cold prevention and management and may not reflect those of multivitamins containing a combination of micronutrients, although the latter is readily available for the general public. Maggini et al.^62^ demonstrated that cold duration and symptom severity was significantly reduced when patients with common cold were provided with a combination of 1,000 mg vitamin C and 10 mg zinc together for 5 days (day 4: *P*_duration_ = 0.01, *P*_severity_ = 0.04). This finding by Maggini et al.^8,62^ highlights that plausible interactions occurring between certain micronutrients when used in tandem may potentially have synergistic, or even antagonistic, effects on cold management.^63^ Thus, significantly different effects on guarding against and treating cold may be observed when a combination of micronutrients are provided, compared with when they were provided alone. In addition, our review does not account for the effects of micronutrients provided via non-oral means, such as through topical creams, nasal gels, or intravenously, some of which presented positive effects on the outcomes of interest in this review.^57,64^ Our selected studies were all conducted in Western temperate countries except for one study in Japan; hence, our results may not extend to populations living in tropical countries or the Asian population. In temperate countries, colds and flu episodes generally occur during the winter, whereas colds and flu episodes usually occur throughout the year in tropical countries.^65^ Therefore, if the same study is conducted in June in the temperate countries and in tropical countries, the chance of colds would be higher in tropical countries than in temperate countries. This could lead to potential confounding effects on the risk effects.^65^ Indeed, to the best of our knowledge, there was no study investigating the association between micronutrient deficiency and cold incidence and/or management in healthy adults living in the tropics. Furthermore, all but one^26^ of our selected studies were conducted with Caucasian population. Given the different host genetic makeup between Caucasian and Asian populations, our review findings may not be generalized to healthy Asian population. In addition, the relatively homogeneous diet in Western population is significantly different than those in Asian population, which tend to consume phytates in higher amounts, therefore influencing zinc absorption.^66–68^ Thus, our results may only be generalizable to healthy Caucasians living in temperate countries.

Another significant limitation is that most cold episodes were self-reported^26,27,29,31–34,40,41,45^ and confirmation of cold resolution by healthcare professionals was lacking in all but five studies.^39–42,45^ Self-reported colds and cold resolution were detected by a change in symptom score in the tools measuring symptom severity. Studies attempted to decrease the risk of detection bias in their self-reporting methods by using validated tools such as the WURSS-21^61^. However, these tools are still liable to selective or inaccurate reporting by subjects. Thus, detection bias is likely to exist in most studies, although we attempted to minimize this bias by extracting data from clinically diagnosed or laboratory-confirmed episodes when available.

Last, there was very low certainty in the results obtained for the efficacy of micronutrient supplementation for cold prevention, and low certainty for the results obtained for the effectiveness of micronutrient administration for cold management was due to the lack of bio-accessibility and bioavailability evidence of the micronutrient assessed in RCT.^48^ In addition, we acknowledge that the small number of studies included in our review may cause inaccurate detection of publication bias in our outcomes of interest. This is especially so when we assessed the outcomes for publication bias by micronutrient singly. At best, we included 11 (cold duration) and nine (cold prevention) studies for our respective outcomes of interest when we pooled all micronutrients reporting that outcome. For a funnel plot to be able to detect publication bias with adequate power, 10 studies are required minimally. Although the number of studies we included for each outcome is close to the minimally required 10 studies, it remains probable that asymmetry may exist because of chance rather than true publication bias.

## CONCLUSION

Overall, the results suggest that micronutrients other than vitamin C may have limited effects on cold prevention among healthy adults, but zinc shows potential reduction of cold duration.

## Supplemental tables

Supplemental materials
