# Supplementary material for: Zinc Supplementation Reduces Common Cold Duration among Healthy Adults: A Systematic Review of Randomized Controlled Trials with Micronutrients Supplementation
Source: Am J Trop Med Hyg. 2020 Apr 27;103(1):86–99. doi: 10.4269/ajtmh.19-0718 (PMC7356429; doi:10.4269/ajtmh.19-0718)
Supplement: Supplementary file 1 [file tpmd190718.SD1.pdf]

Supplemental Materials

Table S1. Example of search strategy for the efficacy of zinc in prevention and management of cold and/or pneumonia PubMED, Embase, Scopus.

**Database**	**Search terms**	**PICOS criteria**	**Results yielded**
PubMED	(Adult OR Middle aged OR Young Adult) NOT (children OR elderly)	Population	[redacted]
	("Zinc Compounds"[Mesh] OR "Zinc"[Title/Abstract]	Intervention	
	("Pneumonia"[Mesh] AND "Community-Acquired Infections"[Mesh]) OR "Respiratory Tract Infections"[Mesh] OR "Community-acquired Pneumonia" OR "respiratory tract infections") NOT "tuberculosis	Outcome	
	((((randomized controlled trial[pt] OR controlled clinical trial[pt] OR randomized[tiab] OR randomised[tiab] OR randomization[tiab] OR randomisation[tiab] OR placebo[tiab] OR drug therapy[sh] OR randomly[tiab] OR trial[tiab] OR groups[tiab]) NOT (animals[mh] NOT humans[mh])))	Study Design	
		PICOS	14
Embase	'adult'/exp	Population	[redacted]
	'zinc'/exp NOT 'nasal'/exp	Intervention	
	('community acquired pneumonia'/exp OR 'common cold'/exp OR 'viral respiratory tract infection'/exp)	Outcome	
	('crossover procedure':de OR 'double-blind procedure':de OR 'randomized controlled trial':de OR 'single-blind procedure':de OR random*:de,ab,ti OR factorial*:de,ab,ti OR crossover*:de,ab,ti OR ((cross NEXT/1 over*):de,ab,ti) OR placebo*:de,ab,ti OR ((doubl* NEAR/1 blind*):de,ab,ti) OR ((singl* NEAR/1 blind*):de,ab,ti) OR assign*:de,ab,ti OR allocat*:de,ab,ti OR volunteer*:de,ab,ti)	Study Design	
		PICOS	15
Scopus	(TITLE-ABS-KEY (adults) AND NOT TITLE-ABS-KEY (children) AND NOT TITLE-ABS-KEY (elderly) AND NOT TITLE-ABS-KEY (pregnan))	Population	[redacted]
	(TITLE-ABS-KEY (zinc) AND NOT TITLE-ABS-KEY (gel) AND NOT TITLE-ABS-KEY (spray))	Interv	

	ention	[redacted]
(TITLE-ABS-KEY (community AND acquired AND pneumonia) OR TITLE-ABS-KEY (common AND cold) OR TITLE-ABS-KEY (respiratory AND tract AND infection) OR TITLE-ABS-KEY (febrile AND respiratory AND illness))	Outcome	
((TITLE-ABS-KEY (randomised AND controlled AND trial) OR TITLE-ABS-KEY (randomized AND controlled AND trial))	Study Design	
	PICOS	30

Table S2. Example of search strategy for the efficacy of zinc in prevention and management of cold and/or pneumonia in the Cochrane Library.

**Database**	**Search ID**	**Search terms**	**PICOS criteria**	**Results yielded**
Cochrane Library	#1	MeSH descriptor: [Adult] explode all trees	Population	[redacted]
	#2	MeSH descriptor: [Young Adult] explode all trees		
	#3	MeSH descriptor: [Middle Aged] explode all trees		
	#4	MeSH descriptor: [Child] explode all trees		
	#5	MeSH descriptor: [Aged] explode all trees		
	#6	(#1 and #2 and #3) not (#4 or #5)		
	#7	MeSH descriptor: [Pneumonia] explode all trees	Outcome	
	#8	MeSH descriptor: [Community-Acquired Infections] explode all trees		
	#9	(#7 AND #8)		
	#10	MeSH descriptor: [Common Cold] explode all trees		
	#11	MeSH descriptor: [Respiratory Tract Infections] explode all trees		
	#12	"respiratory tract infection" or "flu" or "respiratory infection" or "febrile respiratory illness"		
	#13	#9 OR #10 OR #11 OR #12		
	#14	MeSH descriptor: [Dietary Supplements] explode all trees	Intervention	
	#15	supplement* ;ti,ab		
	#16	MeSH descriptor: [Zinc] explode all trees		
	#17	zinc;ti,ab		
	#18	MeSH descriptor: [Zinc Compounds] explode all trees		
	#19	zinc gluconate OR zinc carnosine OR zinc bisglycinate		

#20	MeSH descriptor: [Zinc Acetate] explode all trees		[redacted]
#21	MeSH descriptor: [Orotic Acid] explode all trees		
#22	(#14 OR #15) AND (#16 OR #17 OR #18 OR #19 OR #20 OR #21)		
#23	#16 OR #17 OR #18 OR #19 OR #20 OR #21		
#24	("Zinc Sulfate" or "Zinc Acetate" or "Zinc Oxide" or "Zinc Compounds" or "Zinc" or "Zinc gluconate" or "Zinc Sulfate" or "Zinc Acetate" or "Zinc Oxide" or "Zinc Compounds" or "Zinc orotate" or "Zinc picolinate" or "Zinc carnosine" or "Zinc bisglycinate"):ti,ab		
#25	MeSH descriptor: [Randomized Controlled Trial] explode all trees	Study Design	
#26	#6 and #13 and #23 and #25	PICOS	0
#28	#6 and #13 and #24 and #25		0

Table S3. Details and support for risk of bias assessment

Author, Year [Ref.]	Risk of bias assessment details
**Vitamin A or E (2 studies)**	

Hemila, 2002 [1]

Bias	Authors' judgement	Support for judgement
Random sequence generation (selection bias)	Low risk	Quote: "The participants were randomly assigned..."; "Randomization was performed in blocks of eight within each of the study areas."<br>Comments: Randomisation was likely done
Allocation concealment (selection bias)	High risk	Quote: "Randomization was performed in blocks of eight within each of the study areas."<br>Comment: Block randomisation with fixed block size (of 8) used may not be sufficent to conceal allocation
Blinding of participants and personnel (performance bias)	Low risk	Quote:"...Study was a randomized, double-blind..."<br>Comment: Blinding procedures not described although trial was described to be "double-blinded". Research group seemed to not be blinded to subjects' allocation during the trial. Nonetheless, the blinding is most likely to not influence outcome since the outcome occurrence is a natural onset of illness.
Group Comparability (performance bias)	Low risk	Quote: "There were no essential differences among the randomized groups in the medians or distributions of any of the characteristics examined."<br>Comment: Baseline characteristics in all groups not statistically analysed but median, 20 percentile and 80 percentile values are very similar.
Blinding of outcome assessment (detection bias)	High risk	Quote: "self-reported illnesses were not further verified"<br>Comment: Patient-reported outcome not verified and is prone to recall bias or selective reporting.
Incomplete outcome data (attrition bias)	High risk	Quote: "Of 5,450 placebo group... 280 men were missing data on diet, leaving 5,170 men for the analysis on diet."
Selective reporting (reporting bias)	Unclear risk	Quote: "The events for this study... using the unique personal identification number for linkage"; "Secondary objectives were... incidence of other diseases"<br>Comment: Illness incidence was not specifically pre-specified as secondary outcome, although predefined questions for cold incidence asked during follow-up visits. However, unclear whether primary and other secondary outcomes were determined and reported since study outcomes were split into many different papers.
Other bias	Unclear risk	Recall bias may be present due to patient-reported outcome. However, insufficient information is provided to determine whether it exists.

Hemila, 2004

Bias	Authors' judgement	Support for judgement
[illegible]	[illegible]	[illegible]

[2]

Vitamin D (8 studies)

De Gruijl, 2012 [3]

Bias	Authors' judgement	Support for judgement
Random sequence generation (selection bias)	Low risk	Quote: "Volunteers were randomly assigned to one of the three study groups (using the entry numbers and www.randomizer.org)"<br>Comment: Likely done since randomisation method was described as well
Allocation concealment (selection bias)	Low risk	Quote: "Volunteers were randomly assigned to one of the three study groups (using the entry numbers and www.randomizer.org)"<br>Comment: Computer generated allocation is likely to be unpredictable
Blinding of participants and personnel (performance bias)	High risk	Quote: "Group (B) with 37 volunteers (excluding one drop-out) took daily 1000 IU (25 μg) of vitamin D3 orally in gel capsules...Group (C) was the control group with 33 volunteers who wereasked to "go about their usual business"..."; "from instructions ... the participants knew their assigned groups before filling out questionnaires."<br>Comment: Personnel and subjects were unblinded to treatment allocation due to nature of intervention and the lack of effort to blind subjects in the placebo group.
Group Comparability (performance bias)	Low risk	Quote: Refer to Table 1<br>Comment: No significant differences in baseline characteristics between groups, other than experiencing rases frp, sun exposure. However, this characteristic is unlikely to affect outcome.
Blinding of outcome assessment (detection bias)	Low risk	Quote: "Volunteers were not informed on the main objective of the study, and were told that the aim was to find an optimal method to correct the winter low in vitamin D status."<br>Comment: Preventive measure was taken against selective reporting from subjects, since the outcome was patient-reported.
Incomplete outcome data (attrition bias)	Low risk	Quote: "21 people reported missing a day ... always compensated with an extra capsule the next day."<br>Comment: Missed days are unlikely to have a clinically relevant impact on the cold incidence, especially since the missed days are compensated for the next day.
Selective reporting (reporting bias)	Low risk	Quote:"The legally required permission for this (non-medicinal) study...registered under investigation number P07.035 (amended)."; "We collected blood samples from all volunteers... After the 8-week intervention weinquired about colds."<br>Comment: Trial is registered; Pre-specified outcomes (Measured skin colour in sunbed users, Vitamin D status before and after intervention, cold incidence after 8 weeks) were all reported.
Other bias	Unclear risk	Recall bias might be present since this is patient-reported outcome but insufficient information is provided to decide determine the importance of risk

	Bias	Authors' judgement	Support for judgement
Goodall, 2014 [4]	Random sequence generation (selection bias)	Low risk	Quote: "Participants were then randomized to one of four allocation arms"<br>Comment: Likely done.
	Allocation concealment (selection bias)	Low risk	Quote:"The study sample was stratified based on housing ...block randomization occurred within each stratum using a 1:1:1:1 allocation ratio."; " allocation was concealed using opaque, sealed, serially numbered envelopes"<br>Comment: Highly predictable allocation sequence since block randomisation was done sequentially. However, only the study pharmacist knew the allocation scheme and the envelopes were only accessed by 2 study personnel not involved in the preparation of envelopes and size of randomisation blocks were unknown to study personnel.
	Blinding of participants and personnel (performance bias)	Low risk	Quote:" The study was double-blind with respect to the vitamin D3/placebo intervention... All other participants and study personnel remained blinded."; "Participants were randomized to receive a container with eight capsules of either 10,000 IU of active vitamin D3 or identical placebo"<br>Comment: Likely done due to allocation and blinding techniques used.
	Group Comparability (performance bias)	Low risk	Quote: "Baseline characteristics were similar across the intervention arms (Table 1)."<br>Comment: Bias is unlikely since there are no significant differences in baseline characteristics between groups.
	Blinding of outcome assessment (detection bias)	Low risk	Quote: "Participants were asked to complete weekly online surveys..and to submit one self-collected nasal swab weekly."; "Only swabs submitted from symptomatic participants were tested for respiratory viruses"; "Adjudication by two clinicians was applied when participants reported symptoms but were uncertain if they were ill."<br>Comment: Detection bias unlikely since URTI is laboratory confirmed if reported, and clinically confirmed if unsure.Furthermore, participants were blinded to their allocation outcome
	Incomplete outcome data (attrition bias)	Low risk	Quote: Multiple imputation, using the Markov chain Monte Carlo method, ... Information collected at baseline and through weekly surveys was used to predict missing values..."<br>Comment: Missing data have been imputed using appropriate methods.
	Selective reporting (reporting bias)	Low risk	Quote: "Primary outcome was the incidence of clinical UTRI"; "Secondary outcomes included laboratory confirmed illness, viral load, and symptom duration and severity."<br>Comment: All pre-specified outcomes were reported using the methods described
	Other bias	Low risk	Study appears to be free of other sources of bias.

Laaksi, 2010 [5]

Bias	Authors' judgement	Support for judgement
Random sequence generation (selection bias)	Low risk	Quote:"The subjects were randomly assigned to..." Comment: Likely done since assignment methods were reported
Allocation concealment (selection bias)	Low risk	Quote:"Random allocation was performed using computer-generated random numbers." Comment: Low risk since assignment cannot be foreseen using this method.
Blinding of participants and personnel (performance bias)	Low risk	Quote: subjects were randomly assigned to the intervention group...or the control group (np84), which received placebo (Pharmia; a capsule identical in size and form to the active preparation)" Comment: Subjects were blinded but insufficient information was provided to determine whether ket study personnel were blinded. However, lack of blinding study personnel is unlikely to influence outcome.
Group Comparability (performance bias)	Low risk	Quote: "there was no statistically significant difference in mean serum 25(OH)D concentrations.. Other characteristics were also comparable between the groups at baseline ... (Table 1)." Comment: Low risk since baseline characteristics between groups were similar
Blinding of outcome assessment (detection bias)	Low risk	Quote: "physicians and other personnel treating patients in garrisons were blinded to treatment allocation." Comment: Outcome assessors were blinded and outcomes were obtained from medical records.
Incomplete outcome data (attrition bias)	High risk	Quote: "60 subjects dropped out of the study by the end point with no specific reason given for study withdrawal (Figure 1)."; 21 subjects dropped out of Intervention group and 39 dropped out of placebo group (Figure 1). Comment: Data was still analysed per intention to treat although there was substantial missing outcome data, in inbalanced amounts, across the treatment groups.
Selective reporting (reporting bias)	Low risk	Quote: "The study was registered in ClinicalTrials.gov(NCT00973583)."; "main outcome variable was the number of days absent from duty ... Secondary outcomes were self-reported symptoms of acute respiratory tract infection (cough, runny nose, sore throat, fever, or common cold symptoms) and hospitalization due to acute respiratory tract infection" Comment: All prescpecified outcomes were reported in the publication using reported methods.
Other bias	High risk	Quote: "observed effect was 72% of that size" Comment: sample size was only able to detect a difference in the main outcome between groups with 72% power, suggesting possible risk of reporting bias.

Li-Ng, 2009 [6]

Bias	Authors' judgement	Support for judgement
Random sequence generation (selection bias)	Low risk	Quote: "3-month prospective, randomized, double-blind, placebo-controlled trial of vitamin D3 supplementation" Comment: Likely done since randomisation technique used was reported.
Allocation concealment (selection bias)	Low risk	Quote:"participants were randomly assigned using a computer-generated randomization sequence"; "Each subject was sequentially assigned a number upon study entry...container of study medication to the subject" Comment: Computer-generated randomisation is unpredictable and sequentially bumbered drug containers with identical appearances were used.
Blinding of participants and personnel (performance bias)	Low risk	Quote:"All participants and investigators were blinded throughout the study except for the research pharmacist and the statistician. Neither the statistician nor the research pharmacist had any contact with study participants." Comment: Ample measures taken to blind subjects and key research personnel.
Group Comparability (performance bias)	Low risk	Quote:"There were no significant differences between the active and placebo patients at baseline.", Refer to Table 1 Comment: Low risk since baseline characteristics were similar across groups
Blinding of outcome assessment (detection bias)	Low risk	Quote: "bi-weekly questionnaire ... record the incidence of URI symptoms in the subjects" Comment: Outcomes were patient-reported. However, low risk since subjects were blinded to their treatment allocation and the lack of blinding is unlikely to influence outcome reporting
Incomplete outcome data (attrition bias)	Low risk	Quote: Refer to Fig 1 for number of subjects discontinued and reasons for discontinuing (6 in intervention group, 8 in placebo group); "only results calculated within subjects who actually report a URI using all available data are reported." Comment: numbers of missing outcome data is quite balanced across group and reasons of discontinuation were somewhat similar.
Selective reporting (reporting bias)	Low risk	Quote: "aim of this study was to evaluate whether vitamin D3 supplementation...prevents symptomatic URIs in adults,and ... decreases the severity and duration of URI symptoms."; "Questionnaire inquired about symptoms of URI, duration and severity of symptoms, sick contacts, medication use, sick leave due to illness, and doctor visits."; Serum 25-OHD and serum PTH were measured as well. Comment: All outcomes of interest were pre-specified and reported
Other bias	High risk	There may be a high risk of detection bias since illness is not confirmed through clinical or laboratory means.Study was also underpowered.

Study	Bias	Authors' judgement	Support for judgement
Murdoch, 2012 [7]	Random sequence generation (selection bias)	Low risk	Quote:" Participants were assigned using computer- generated randomization to receive either vitamin D3 or placebo."<br>Comment: Likely done since technique for randomisation was reported
	Allocation concealment (selection bias)	Low risk	Quote:" Participants were assigned using computer- generated randomization to receive either vitamin D3 or placebo."<br>Comment: computer-generated randomisation is unpredictable, allowing allocation to be concealed
	Blinding of participants and personnel (performance bias)	Low risk	Quote: "those randomised to placebo received matching inactive tablets"; "randomization process and bottling of tablets were performed in Auckland, New Zealand,..to ensure that those running the study, including outcome assessors and those administering the intervention, were blinded to allocation."<br>Comment: Measures were taken to blind subjects and ket research personnel to allocation of subjects
	Group Comparability (performance bias)	Low risk	Quote: "The groups were evenly balanced on all characteristics"<br>Comment: No differences between baseline characteristics scross groups
	Blinding of outcome assessment (detection bias)	Low risk	Quote: "outcome assessors and those administering the intervention, were blinded to allocation."<br>Comment: Blinding measures were taken and it was unlikely that blinding was broken due to geographical proximity of where study was conducted, relative to where bottling was conducted.
	Incomplete outcome data (attrition bias)	Low risk	Quote: "missing observations were estimated using ... Markov chain Monte Carlo method on natural log scores ..."; quite balanced number of missing data in both groups, with similar reasons (FIg 1).<br>Comment: Although missing outcome data were quite balanced in numbers across intervention groups, the data was analysed with intention-to-treat. Nevertheless, missing data have been imputed with appropriate methods, thus risk of bias is low.
	Selective reporting (reporting bias)	Low risk	Quote:"The primary end point was number of URTI episodes. Secondary end points were number of days of missed work as a result of URTI episodes, duration of URTI episodes, severity of URTI episodes, and detection of respiratory viruses in nasopharyngeal samples."; "Plasma calcium and serum 25-OHD levels were measured at baseline and at 2, 6, 12, and 18 months after enrollment"<br>Comment: All prespecified outcomes were reported using methods described
	Other bias	Low risk	There appears to be no other source of bias

Rees, 2013 [8]

Bias	Authors' judgement	Support for judgement
Random sequence generation (selection bias)	Low risk	Quote:".. a web-based, random number generator assigned treatment within blocks, stratified by study center, sex, and colonoscopy interval (3 or 5 years)..."<br>Comment: Likely done since randomisation process was reported
Allocation concealment (selection bias)	Low risk	Quote:".. a web-based, random number generator assigned treatment within blocks, stratified by study center, sex, and colonoscopy interval (3 or 5 years)..."<br>Comment: web-based random number generator is unpredictable
Blinding of participants and personnel (performance bias)	Low risk	Quote:"Participants and investigators were blinded;the computer programming staff, 1 pharmacy technician, statistician, and statistical analyst were unblinded."; "identical-looking pills containing vitamin D3, calcium carbonate, both, or placebo"<br>Comment: Measures were taken to blind subjects and key researc personnel
Group Comparability (performance bias)	High risk	Quote: No significant differences except pretrial Vit C intake and serum 25(OH)D status between groups are significantly different (Refer to Figure 1); "effect of vitamin D supplementation on episodes or days of illness was not significantly modified by 25(OH)D status at enrollment."; "In an observational analysis, the risk of URTI based on quartiles of serum 25(OH)D suggested a significantly higher risk of ILI (but not colds) in the lowest quartile of serum 25(OH), ...not including randomized treatment (Table 3)."<br>Comment: Some significant differences in baseline characteristics between groups which may influence some outcomes. This is especially since vitamin D status was significantly different at baseline.
Blinding of outcome assessment (detection bias)	Low risk	Quote:"completing daily health diaries regarding fever, headache, muscle aches, chills, cough, runny nose, and allergies"<br>Comment: Patient-reported outcomes are unlikely to be influenced by lack of blinding since they are already blinded to their treatment group.
Incomplete outcome data (attrition bias)	Low risk	Quote: Sensitivity analyses to explore effects of missing data..are described in the Supplementary Appendix; "None of our approaches to assess the impact of missing data, ... appreciably altered the effect estimates or yielded statistically significant associations between vitamin D_3 supplementation and URTI symptoms"<br>Comment: Missing data did not have plausible effect size to have a clinically relevant impact on observed effect size.
Selective reporting (reporting bias)	Unclear risk	Quote:"we investigated whether vitamin D3 supplementation (1000 IU/day) would reduce the number of episodes and duration of URTI in winter and throughout the year, and the number of episodes and duration of winter ILI and of colds"<br>Comment: All prespecified outcomes were reported using described methods. However, there were additional results (effect of calcium supplementation on incidence and duration on URRI incidence and duration, results are most likely from parent study) which was reported without showing full data.
Other bias	High risk	Insufficient information to asses whether an important risk of bias exists e.g. selecting participants from a parent study may be influenced by early effect of study treatment on URTI symptoms. However, detection bias is likely to be present due to lack of laboratory confirmation of URTI and and potential misclassification of colds and ILI by symptom-based case definition

Study	Bias	Authors' judgement	Support for judgement
Shimizu, 2018 [9]	Random sequence generation (selection bias)	Low risk	Quote:"staff who has no involvement in the study prepared an assignment list using random numbers, ... were given to the two groups respectively"<br>Comment: likely done since randomisation methods were described
	Allocation concealment (selection bias)	Unclear risk	Quote:"staff who has no involvement in the study prepared an assignment list using random numbers, ... were given to the two groups respectively"<br>Comment: Randomisation is done but more details is required to determine predictability of "randomly divided" method used by the staff who decided allocation.
	Blinding of participants and personnel (performance bias)	Unclear risk	Quote:"The subjects for blinding were all those who were involved in the study...unblinded after securing analysis subjects"; "Placebo was prepared by...so that it would not be distinguished from the trial supplement by the color"<br>Comment: Measures were taken to blind subjects and key research personnel. However, more information is needed to determine extent of research personnel blinding.
	Group Comparability (performance bias)	Low risk	Quote: "the subjects who were determined to be in the deficiency state...the difference was not significant between the two groups (P = 0.891)."<br>Comment: Baseline characteristics across groups were similar
	Blinding of outcome assessment (detection bias)	Low risk	Quote:"subjects were required to fill in the WURSS-21 and to record subjective symptoms"<br>Comment: Outcome assessors (patients themselves) are blinded since outcomes are patient-reported
	Incomplete outcome data (attrition bias)	Low risk	Quote: Refer to Figure 1; "FAS had 125 cases in the 25OHD and 123 cases in the placebo"; "110 cases in the 25OHD and 105 cases in the placebo were in the<br>PPS."<br>Comment: Attrition was quite balanced across intervention groups with similar reasons for attrition.
	Selective reporting (reporting bias)	Low risk	Quote:"The primary outcome measure was the incidence proportion of URTI for the period of 16 weeks of supplement intake"; "secondary outcome measures were the physical severity score, the QOL score, the duration of URTI, and the incidence of new URTI every four weeks."; "As an exploratory efficacy analysis not listed in the protocol, the total physical severity score and the total QOL score were assessed"<br>Comment: All primary and secondary outcomes were reported using prespecified methods. Although an addiitonal outcome was reported as part of exploratory efficacy analysis, the risk of bias is deemed as low dsince the outcome was reported completely with data that can be entered in a meta-analysis.
	Other bias	High risk	Quote: "number of cases in this study was too small to statistically significantly prove the efficacy of 25OHD intake"<br>Comment: Sample size is too small to be able to detect a difference in the main outcome between groups with at least 80% power. Also, there is no clinical or laboratory confirmation for onset of URTI. Hence there is high risk for detection bias

Simpson, 2015 [10]

Bias	Authors' judgement	Support for judgement
Random sequence generation (selection bias)	Low risk	Quote: " Participants were randomised simply 1:1 to parallel treatment using a computerised randomisation program (www.randomization.com), ..."<br>Comment: Likely done since randomisation methods were reported
Allocation concealment (selection bias)	Low risk	Quote: " Participants were randomised simply 1:1 to parallel treatment using a computerised randomisation program (www.randomization.com), ..."<br>Comment: Computer geneated randomisation is unpredictable, hence deemed to be of low risk
Blinding of participants and personnel (performance bias)	Low risk	Quote:"A person outside the study was asked to run the randomisation and to affix the treatment labels with study IDs to the respective bottles of treatment and placebo."; "Both cholecalciferol and placebo were identical white capsules"; "all CIPRIS staff (including nurses and database entry personnel), investigators and participants were blinded to treatment allocation until the conclusion of follow-up."<br>Comment: Measures were taken to ensure blinding of subjects and research personnel
Group Comparability (performance bias)	Low risk	Quote:"None of the cohort characteristics were significantly different between treatment arms."; Refer to Table 1<br>Comment: SImilar baseline characeristics across treatment groups
Blinding of outcome assessment (detection bias)	Low risk	Quote:"Participants completed daily online questionnaires querying the occurrence and magnitude...nonspecific symptoms";"participant was invited to come into clinic for objective assessment by our study nurse."; "infection reports from daily online surveys, and from clinic assessments,were reviewed by the chief investigator and the study nurse,.."<br>Comment: Outcome assessors at all levels (Patients, lab & clinic personnel) were blinded sufficiently
Incomplete outcome data (attrition bias)	Unclear risk	Quote: "For all instances where data was missing, analyses were restricted to persons with complete data"; "Two participants (both on treatment) dropped out during the<br>study ...were replaced with an additional two participants who ran out their period of follow-up."; "Analysis was by intention-to-treat."<br>Comment: Study did not report any effort to impute missing data but performed intention-to-treat analyses although it mentioned that analyses were restricted to people with complete data when data was missing. -> Contradictory statement since dropouts (2 from treatment group) happened fairly early in the intervention (Weeks 1 and 4).
Selective reporting (reporting bias)	Low risk	Quote: "Primary outcomes were time to infection. Secondary outcomes were infection severity and duration. Tertiary outcomes were change in serum 25(OH)D and the occurrence of adverse events.<br>Comment: All outcomes were reported with prespecified methods, hence low risk of bias
Other bias	Unclear risk	Quote: "By relying on participants to report symptoms via daily questionnaire, the subjective<br>nature of what is a reportable symptom is still a limitation."; "The high frequency of infections during the study may indicate that some of the infections reported, particularly those not assessed in clinic, were not true infections"<br>Comment: Suggests the possible likelihood of detection bias, however, insufficient information is provided to assess whether it exists.

Zinc (10 studies)

Douglas, 1987 [11]

Bias	Authors' judgement	Support for judgement
Random sequence generation (selection bias)	Unclear risk	Quote: "manufacturers provided 150 sequentially numbered bottles, each of which contained 48 effervescent tablets of either zinc acetate or placebo randomly allocated to the sequence"<br>Comment: Likely done. However, insufficient information provided to determine risk of bias since sequence generation method was not disclosed.
Allocation concealment (selection bias)	Low risk	Quote: "manufacturers provided 150 sequentially numbered bottles, each of which contained 48 effervescent tablets of either zinc acetate or placebo randomly allocated to the sequence";"It was a double-blind study and all observers remained blind to the identity of the treatment courses until all data were collected."<br>Comment: Probably done since sequentially numbered drug containers of identical appearance were used to conceal allocation and no one knew the identity of the treatment courses
Blinding of participants and personnel (performance bias)	Low risk	Quote: "all observers remained blind to the identity of the treatment courses until all data were collected"<br>Comment: Likely done
Group Comparability (performance bias)	Low risk	Quote: The durations of medication in the two groups were similar, ...Generally, ..., the comparibility of the two treatment groups was acceptable."<br>Comment: Baseline characteristics accross treatment groups were similar
Blinding of outcome assessment (detection bias)	Low risk	Quote: "It was a doubleblind study and all observers remained blind to the identity of the treatment courses until all data were collected. ...for the entire study, it was broken in November."<br>Comment: Patient and nurses were blinded, hence low risk of bias
Incomplete outcome data (attrition bias)	High risk	Quote: "Of the treatment courses, 35 were zinc and 35 were placebo."; "excluded from the analysis treatment courses in which the residual tablet counts indicated that the individual had not used the tablets at least for 3 days and at the rate of 4 or more per day... seven courses were excluded as unevaluable (two zinc and five placebo recipients)."<br>Comment: thrice as much attrition in placebo group (~15%) compared to zinc group (~5%), and reason for attrition (lack of compliance to protocol) could be related to the true outcome
Selective reporting (reporting bias)	Unclear risk	Quote:"For each family, one individual was appointed to supervise the maintenance of symptom diaries";Refer to Table 2<br>Comment: Unclear as to what symptoms were included in the symtom diaries. More information needed to determine if severity scores for systemic symptoms have been ommited from the publication.
Other bias	High risk	Specific study design has a potential risk of detection bias as it was not mentioned if all illness onset is confirmed clinically or laboratory. More information is needed to determine the risk of biasness.

Eby, 1984 [12]

Bias	Authors' judgement	Support for judgement
Random sequence generation (selection bias)	Unclear risk	Quote:"A 7- day supply of tablets (active or placebo) was given to each subject, using a double-blind, random method."<br>Comment: Insufficient information given to determine method of seqeunce generation.
Allocation concealment (selection bias)	Unclear risk	Quote:"A 7- day supply of tablets (active or placebo) was given to each subject, using a double-blind, random method."<br>Comment: Insufficient information given to determine method of allocation and hence, whether allocation was concealed
Blinding of participants and personnel (performance bias)	Unclear risk	Quote: "We used unflavored zinc gluconate tablets ...with matching placebos"; "Both tablets ..were otherwise identical"; "A 7- day supply of tablets (active or placebo) was given to each subject, using a double-blind, random method."<br>Comment: Extensive effort have been made to blind subjects. However, insufficient information is provided to determine whether research personnel were blinded as well.
Group Comparability (performance bias)	Unclear risk	Quote: "The placebo-treated group initially had significantly more severe colds than the zinc-treated group,... initial severity (and initial number of symptoms) had virtually no effect on the duration of colds studied here. The frequency of the 10 symptoms (Table 2) was similar in both groups"<br>Comment: Important baseline characteristics between groups were similar, or had no effect on outcome measure if significantly different. However, initial severity is known to affect cold duration
Blinding of outcome assessment (detection bias)	Low risk	Quote: Subjects recorded the presence and severity of 10 common cold symptoms on a report form. "<br>Comment: Dection bias unlikely as patients, the outcome assessors, were blinded to their treatment group.
Incomplete outcome data (attrition bias)	High risk	Quote: Refer to Table 1-> 37 cases in Zinc, 28 cases in Placebo due to limiting analysis to subjects who reported being ill for 3 days or less before starting experiment; "slightly higher dropout rate in the zinc subjects (23%) compared with the placebo subjects (15%) was probably due to side effects of zinc"; "Among the 65 subjects, 4 in the zinc group and 8 in the placebo group reported that they stopped taking lozenges prematurely...they are included in this report to maximize subjects and to treat both groups equally."<br>Comment: Imbalance in numbers across intervention groups due to reason likely to be related to true outcome. Also, missing outcomes were included in analysis wven though there was no effort to impute the missing data with appropriate methods.
Selective reporting (reporting bias)	Low risk	Quote: "Subjects recorded the presence and severity of 10 common cold symptoms on a report form. ... Symptoms were scored ...Subjects also recorded side effects or complaints and any deviation from the protocol."<br>Comment: All outcomes were reported with prespecified methods.
Other bias	High risk	High dropout rate, and significantly differential dropout rate between groups is likely to result in attrition bias

Godfrey, 1992 [13]

Bias	Authors' judgement	Support for judgement
Random sequence generation (selection bias)	Low risk	Quote: "Randomization by a third party was used to assign the 87 participants to treatment groups. A pharmacist, using a randomization table provided by the study statistician..."<br>Comment: Likely done since method of randomisation was reported.
Allocation concealment (selection bias)	Low risk	Quote: "A pharmacist, using a randomization table provided by the study statistician, packaged containers for individual subjects with lozenges according to the production<br>run number and subject identification number."<br>Comment: Low risk of bias since the allocation was done solely by pharmacist who was also blinded to treatment allocation.
Blinding of participants and personnel (performance bias)	Low risk	Quote:"ZGG lozenges, which were prepared in the same boiled candy base as the<br>placebo contained..";"Patients, investigators and the pharmacist were, therefore, all blinded as to which treatment individual patients had received."<br>Comment: Measures were taken to blind subjects and research personnel.
Group Comparability (performance bias)	Low risk	Quote: "The mean number of days that the patients had experienced symptoms prior to entering the programme was 1.34 days, the same mean for both groups. There was no significant (P < 0.05) difference between the ZGG and placebo treatment groups ..."<br>Comment: Key baseline characteristics were similar across groups.
Blinding of outcome assessment (detection bias)	Low risk	Quote:"Patients, investigators and the pharmacist were, therefore, all blinded as to which treatment individual patients had received."<br>Comment: Measures were taken to blind patients, the outcome assessors, to their treatment assignment.
Incomplete outcome data (attrition bias)	Low risk	Quote: "A total of eight ZGG- and six placebo-treated patients withdrew from the trial ...Illnesses that resulted in patients withdrawing from the study were as follows: two patients had bronchitis and one had viral gastroenteritis in the placebo treatment group; and<br>there was one patient with influenza and one with a bacterial infection in the ZGG treatment<br>group.Other reasons for withdrawing were: ... efficacy doubted by the patient (one ZGG- and one placebotreated patient)... "<br>Comment: Quite balanced numbers of missing outcome data in both groups (19% in ZGG group, 14% in Placebo group), due to reasons that were either not related to the true outcome, or were balanced if they were possibly related to the true outcome (e.g. efficacy doubted)
Selective reporting (reporting bias)	Unclear risk	Quote:"...test treatment effect and immediacy of treatment..."; "patients kept diaries recording the severity of their symptoms... in addition, were asked to record any side effects"<br>Comment: Sufficient information about parameters of "Efficacy" were not given to determine risk of selective reporting.
Other bias	Unclear risk	Insufficient information about statistical power of this study is provided to determine risk of detection bias.

Mossad, 1996 [14]

Bias	Authors' judgement	Support for judgement
Random sequence generation (selection bias)	Low risk	Quote: "statistical consultant prepared a computer-generated randomization code and the packages of medication"<br>Comment: Likely done since randomisation techniques were described.
Allocation concealment (selection bias)	Low risk	Quote: "statistical consultant prepared a computer-generated randomization code and the packages of medication"<br>Comment: Low riisk since allocation was generated by computer
Blinding of participants and personnel (performance bias)	Unclear risk	Quote: "weight, appearance, flavoring content, and texture."; "packages were identical in appearance except for the randomization numbers. The study medication was distributed by the study nurse, who was masked to treatment assignments"<br>Comment: Measures were taken to blind research personnel and subjects.
Group Comparability (performance bias)	Unclear risk	Quote: "The mean (± SD) and median symptom scores at baseline (the first measurement) were ... 9.3 ± 3.6 and 8 for the placebo group, and 7.9 ± 2.8 and 8 for the zinc group. In practice, an increase in score from 8 to 9 entails scoring one symptom one grade higher or developing another mild symptom."; "incidence of individual symptoms at baseline<br>was similar in the two groups for all but two symptoms..."<br>Comment: Key baseline characteristics were likely to be different across groups. However, we do not know whether the groups were statistically different at baseline
Blinding of outcome assessment (detection bias)	Low risk	Quote:"Patients were asked to complete a daily log documenting the severity of symptoms and the medications taken throughout the duration of their cold for as long as 18 days"<br>Comment: Measures were taken to blind the patients, i.e. outcome assessors, from treatment assignment.
Incomplete outcome data (attrition bias)	Low risk	Quote: "Seventeen of the 100 patients (10 in the zinc group and 7 in the placebo group) were considered nonadherent. ... When data were analyzed after these 17 nonadherent patients were excluded, the study conclusions remained the same."<br>Comment: Missing data from the nonadherent subjects did not have a clinically relevant impact on the intervention effect estimate.
Selective reporting (reporting bias)	Low risk	Comment: All prespecified outcomes were reported with methods described.
Other bias	High risk	Quote:"15 patients (10 placebo recipients and 5 zinc recipients) took other cold medications during the study (P = 0.17)."<br>COmment: Potential performance bias may exist since subjects were exposed to other interventions which could influence true outcome. However, intention-to-treat analysis was still used to evaluate outcomes

Petrus, 1998 [15]

Bias	Authors' judgement	Support for judgement
Random sequence generation (selection bias)	Unclear risk	Quote:"this was a randomized, double-masked study"<br>Comment: Insufficient information provided to determine risk of biasness since randomisation method is not reported
Allocation concealment (selection bias)	Unclear risk	Quote:"this was a randomized, double-masked study"<br>Comment: Insufficient information provided to determine risk of biasness since treatment allocation method is not reported
Blinding of participants and personnel (performance bias)	Unclear risk	Quote: "individuals would receive zinc lozenges and some would receive placebo.", "the placebo and zinc lozenges were peppermint flavored."<br>Comment: Measures were taken to blind subjects, but insufficient information provided to determine whether research personnel were really blinded as the study stated in the title.
Group Comparability (performance bias)	Unclear risk	Quote: "Chi-square tests showed no significant associations between treatment group membership and sex, race/ethnicity, and allergy test status. An independent groups t test showed no significant difference in mean age"<br>Comment: Baseline characteristic similar across groups. However, there is insufficient information about severity of illness and number of symptoms at entry (which can affect outcome) at baseline.
Blinding of outcome assessment (detection bias)	Low risk	Quote: "Subjects were also informed that they were required to rate and record their symptoms in a diary at the same time each day"<br>Comment: Outcomes were patient reported. Since patients were likely to be blinded, risk of bias is low.
Incomplete outcome data (attrition bias)	Low risk	Quote: "Only 1 subject was lost to follow-up, and none of the remaining 101 subjects discontinued because of side effects from the lozenges."<br>Comments: Missing data due to subject who dropped out was not included in analysis. Nevertherless, the effect size from missing data is too small to have a clinicallt relevant impact on observed impact size
Selective reporting (reporting bias)	Unclear risk	Quote:"Independent groups t tests were used to test for differences between the zinc and placebo groups in mean age, mean number of days with symptoms, and mean symptom severity ratings."; "ANOVA) procedures were used to determine whether the mean number of days with symptoms or mean symptom severity ratings differed with respect to treatment group and allergy test status considered simultaneously"<br>Comment: All prespecified outcomes were reported using described methods. However, more information is needed to determine how symptom severity was calculated
Other bias	High risk	No form of verification (either clinical or laboratory) was done for resolution of cold. Detection bias possibly exists

Prasad, 2000 [16]

Bias	Authors' judgement	Support for judgement
Random sequence generation (selection bias)	Unclear risk	Quote: "A research consultant prepared the randomisation code ..." Comment: Insufficient information to determine risk since method of code generation was not described.
Allocation concealment (selection bias)	Unclear risk	Quote: "A research consultant prepared...the packages of medication. The packages were identical in appearance... research assistant who was blinded to treatment assignments distributed the study medication" Comment: Insufficient information about allocation method to determine risk of bias
Blinding of participants and personnel (performance bias)	Low risk	Quote:"Placebo and zinc lozenge were identical in weight, appearance, flavour and texture."; "research assistant who was blinded to treatment assignments distributed the study medication"; "None of these percentages exceeded 50%, indicating that blinding was adequate at the outset and was maintained throuout the study" Comment: Measures were taken to blind subjects and research personnel.
Group Comparability (performance bias)	High risk	Quote: Table 1 for demographic characterisitics, plasma zinc levels were normal in both groups" (healthy controls and study participants with colds before grooup assignment); "At baseline, the average severity score was higher in the zinc group than in the placebo group (10.8 vs 8.9)" Comment: Although measures have been taken to ensure that cold duration was similar acorss groups, cold severity which could affect the illness duration was not balanced across groups.
Blinding of outcome assessment (detection bias)	Low risk	Quote:"Participants..complete a daily log documenting the severity of symptoms and the medications taken throughout the duration of the cold"; "participants returned to the clinic for the final visit within 1 day of resolution of cold symptoms... to confirm that cold symptoms had resolved" Comment: Patients were blinded, while most outcomes were patient-reported. The reviewer assumes that cold resolution was confirmed by research personnel, who were blinded to treatment assignment.
Incomplete outcome data (attrition bias)	Low risk	Quote:"Two participants in the placebo group dropped out on day 2. We therefore had complete data on 48 participants" Comment: Reason for dropout not reported, however plausible effect size from missing outcomes is not enough to have a clinicallt relevant impact on observed effecct size.
Selective reporting (reporting bias)	Low risk	Quote: "Primiary end point was the average duration of cold symptoms. Secondary end points were plasma levels of zinc and proinflammatory cytokines";" complete a daily log documenting the severity of symptoms and the medications taken throughout the duration of the cold"; "to assess side effects of the treatment...and mouth irritation" Comment: All prespecified outcomes were reported using described methods.
Other bias	Low risk	Study appears to be free of other sources of bias. (Population size is huge enought to detect difference between groups with at least 80% power; cold incidence/resolution is confirmed through clinical or laboratory means)

Study	Bias	Authors' judgement	Support for judgement
Prasad, 2008 [17]	Random sequence generation (selection bias)	Unclear risk	Quote: "A research consultant prepared the randomisation code ..." Comment: Insufficient information to determine risk since method of code generation was not described.
	Allocation concealment (selection bias)	Unclear risk	Quote: "A research consultant prepared...the packages of medication. The packages were identical in appearance... research assistant who was blinded to treatment assignments distributed the study medication" Comment: Insufficient information about allocation method to determine risk of bias
	Blinding of participants and personnel (performance bias)	Low risk	Quote:"Placebo and zinc lozenge were identical in weight, appearance, flavour and texture."; "research assistant who was blinded to treatment assignments distributed the study medication"; "From these data, we concluded that the binding of the subjects was adequate" Comment: Measures were taken to blind subjects and research personnel.
	Group Comparability (performance bias)	Unclear risk	Quote: Table 1 for demographic charactersitics, "At baseline, the average severity scores for the zinc and placebo groups were 8.32 and 7.78" Comment: Although measures have been taken to ensure that cold duration was similar acorss groups, cold severity which could affect the illness duration may not be balanced across groups (statisitcal significance not reported). More information is needed to assess risk of bias.
	Blinding of outcome assessment (detection bias)	Low risk	Quote:"Participants..complete a daily log documenting the severity of symptoms and the medications taken throughout the duration of the cold"; "participants returned to the clinic for the final visit within 1 day of resolution of cold symptoms... to confirm that cold symptoms had resolved" Comment: Patients were blinded, while most outcomes were patient-reported. The reviewer assumes that cold resolution was confirmed by research personnel, who were blinded to treatment assignment.
	Incomplete outcome data (attrition bias)	Unclear risk	Comment: there was no missing outcome data
	Selective reporting (reporting bias)	Low risk	Quote: "Our primary end point was the average duration of cold symptoms. Secondary end points were plasma levels of (1) zinc; (2) soluble interleukin (IL)-1 receptor antagonist (sIL-1ra) and soluble tumor necrosis factor (TNF) receptor (sTNF-R) 1; and (3) the plasma adhesion molecules, soluble vascular endothelial cell adhesion molecule (sVCAM)-1 and soluble ICAM (sICAM)-1" Comment: All prespecified outcomes were reported using described methods.
	Other bias	Low risk	Study appears to be free of other sources of bias (Population size is huge enought to detect difference between groups with at least 80% power; cold incidence/resolution is confirmed through clinical or laboratory means)
Turner, 2000 [18]	Random sequence generation (selection bias)	Unclear risk	Quote: " Subjects who met the criteria for randomization to treatment were randomly assigned to 1 of the 4 treatments in accordance with the drug-randomization code. Comment: Likely done but insufficient information to determine the risk of biasness since sequence generation method was not described.
	Allocation concealment (selection bias)	Unclear risk	Quote: " Subjects who met the criteria for randomization to treatment were randomly assigned to 1 of the 4 treatments in accordance with the drug-randomization code. Comment: Iinsufficient information to determine the risk of biasness since allocation method was not described.
	Blinding of participants and personnel (performance bias)	Low risk	Quote: "investigator and the subject were blinded to the identification of the test medications, the study medications were not matched for appearance, flavor, content, and texture."; "responses of the volunteers suggest...study was adequately blinded" Comment: Measures taken to blind patients and research personnel were sufficient.
	Group Comparability (performance bias)	Low risk	Quote: "No differences were noted in the demographic characteristics of the subjects randomized to the different treatment groups. The mean total symptom scores (5 SEM) at the start of study-medication administration were 6.34 (.39) in the placebo group, 6.7 (.44) in the zinc gluconate group, 6.3 (.40) in the 5-mg zinc acetate group, and 6.9 (.38) in the 11.5-mg zinc acetate group (P= .448)." Comment: Key baseline characteristics which could affect outcomes were similar across groups.
	Blinding of outcome assessment (detection bias)	Low risk	Quote: "Illness severity was assessed by subjective symptom scores...After randomization, the symptom scores were recorded by the subject at »12-h intervals" Comment: Likely blinded since patients (the outcome assessors) were blinded and outcomes were patient reported.
	Incomplete outcome data (attrition bias)	Unclear risk	Quote: "data were assessed in an intent-to-treat analysis that included all subjects randomized to treatment"; "Two-hundred eighty-one subjects were randomized to receive 1 of the 3 treatments in the natural colds study" (but summing up subject numbers in Figure 1B only gave 279 subjects) Comment: Reasons behind missing outcome data was not given and the study did not mention anything about missing outcome data. More information is needed to determine the risk of biasness
	Selective reporting (reporting bias)	Low risk	Quote: "primary efficacy analyses in both studies were the comparisons of the durations of cold symptoms in subjects treated...received placebo"; "Symptom severity was also analyzed as a secondary end point." Comment: Primary and secondary outcomes were reported as prespecified.
	Other bias	High risk	No form of confirmation for cold resolution, either through lab or clinical means. Thus there is possible risk of detection bias

	Bias	Authors' judgement	Support for judgement
Veverka, 2000 [19]	Random sequence generation (selection bias)	High risk	Quote: "Randomization was accomplished by using the last number of each cadet's social security in an odd/even fashion (even were assigned to the zinc group, odd were assigned to the placebo). Those with a social security number ending in zero were assigned based on the second to last odd/even number"<br>Comment: High risk since randomisation is inadequate, especially if the social security number is generated using a non-random approach.
	Allocation concealment (selection bias)	High risk	Quote: "Randomization was accomplished by using the last number of each cadet's social security in an odd/even fashion (even were assigned to the zinc group, odd were assigned to the placebo). Those with a social security number ending in zero were assigned based on the second to last odd/even number"<br>Comment: High risk since concealment is inadequate and allocation is predictable once social security number is known.
	Blinding of participants and personnel (performance bias)	Low risk	Quote: "Practitioners or individuals involved in drawing blood or running laboratory analyses were also blinded to the assignment."; "Both zinc (15 mg per capsule) and placebo capsules (same capsule type as used for the zinc except filled with cornstarch or gelatin) were provided in a 30-day supply container"<br>Comment: Measures were taken to blind subjects and research personnel. Review authors believe that measure taken were sufficient to blind the subjects and personnel
	Group Comparability (performance bias)	Low risk	Quote: "Neither ages of the two groups differed significantly"; "no subjects claimed to have a significant medical history or to be under the care of a health care provider for a health concern."<br>Comment: Key baseline characteristics between groups are similar.
	Blinding of outcome assessment (detection bias)	Low risk	Quote: "Practitioners or individuals involved in drawing blood or running laboratory analyses were also blinded to the assignment."; "we reviewed the results of a secure weekly Internet survey where subjects reported their health status."<br>Comment: Subjects and physicians were blinded, thus low risk.
	Incomplete outcome data (attrition bias)	Unclear risk	Quote: "Of 40 subjects at onset of study, 10 dropped from the study through self elimination.Of these, only two subjects within the placebo group complained of gastric discomfort and slight nausea."; "At the conclusion of the study, each group had five subjects that were lost to follow-up"<br>Comment: Balanced numbers of missing outcome data across groups, however not all reasons for missing outcomes were reported. Hence more information is needed to assess risk of bias
	Selective reporting (reporting bias)	Unclear risk	Quote: "primary objective for the study was URI infection rate, we also tracked the results of the laboratory analyses for plasma zinc and plasma copper levels"; "The purpose of the secure Internet survey was to capture data on individuals who may not have felt ill enough to see a medical provider but was still experiencing some overt symptoms associated with URIs"<br>Comment: All outcomes were reported with prespecified methods. However, severity scores were collected but not reported. More information is needed to determine the purpose the severity scores were collected and hence, risk of bias.
	Other bias	High risk	Quote: "A total sample size of 34 was derived based on a 25% reduction in URI incidence rates detected in the zinc supplemented group with a 2 sided p value of 0.05 and an approximate power of 80%."; "Limitations for this study included self report data derived from the online web survey, higher than anticipated drop-out rate and a lower than desired response rate to the web survey."<br>Comment: Sample size is too small to detect difference between group with sufficient statistical power due to higher than anticipated dropout rate. Also, all mentioned limitations contribute to potential detection bias. Hence, there is a risk for detection bias

Study	Bias	Authors' judgement	Support for judgement
Weismann, 1990 [20]	Random sequence generation (selection bias)	Unclear risk	Comment: Trial was not described as a randomised trial, method of random sequence generation was not reported and more information is needed to determine risk of biasness.
	Allocation concealment (selection bias)	Unclear risk	Comment: Trial was not described as a randomised trial, method of allocation was not reported and more information is needed to determine risk of biasness.
	Blinding of participants and personnel (performance bias)	Unclear risk	Quote: "The study was designed as a prospective double-blind clinical trial"; "The zinc and placebo lozenges consisted of maltitol syrup with natural flavours"<br>Comment: Probably done but more information is needed to permit judgement that blinding of subjects and personnel was performed, which could affect outcomes reported.
	Group Comparability (performance bias)	Low risk	Quote:"No statistically significant difference at 5% level between the two groups with regard to sex, age, smoking or severity of symptoms at the start of the study was present. There was no statistically significant difference between treatment groups with respect to the frequency of patients who did not experience a cold and hence did not participate in the study (cf. Table 1)."<br>Comment: Key characteristics at baseline (which could affect outcomes measured) were similar across groups.
	Blinding of outcome assessment (detection bias)	Unclear risk	Quote: "Ten days after the start of the trial, all patients were to consult their physician..."; "At the start of the study, the patients registered the following symptoms...A schedule indicating many, some, or no symptoms was filled out. During the following days, the patients were instructed to note their overall condition every evening"<br>Comment: More information is needed to assess whether physicians and subjects (main outcome assessors) were blinded during the study.
	Incomplete outcome data (attrition bias)	High risk	Quote: Refer to Table 1 -> Number of patients excluded due to missing records:8/77 (10%, placebo), 6/68 (9%, zinc); Number of patients excluded due to too low age: 1/68 (zinc)<br>Comment: Attrition rate for same reason is similar across groups, but could have a plausible effect size to induce clinically relevant bias in observed effect size.
	Selective reporting (reporting bias)	High risk	Quote: "A schedule indicating many, some, or no symptoms was filled out. During the following days, the patients were instructed to note their overall condition every evening"; "Side-effects were noted and specified in the diary"<br>Comment: Study reports prespecified outcomes (cold duration and severity) using reported methods. Additional outcomes, such as the comparison of the course of actual common cold with prior episodes, difference between number of tablets taken during study between the 2 groups, as well as the statistical analysis of side effects, were reported although not prespecified.
	Other bias	Unclear risk	Statistical power of study was not reported, and the cold episode not clinically or laboratory confirmed (visit to physician may or may not be able to confirm the incidence of cold since some may recover quickly). More information is needed to determine the risk of detection bias in this study.

Table S4. GRADE evidence profile

Certainty assessment							No. of patients		Effect		Certainty
No. of studies	Study design	Risk of bias	Inconsistency	Indirectness	Imprecision	Other considerations	micronutrients	placebo	Relative (95% CI)	Absolute (95% CI)	
Preventing Cold/ARI episodes (assessed with: Vitamin D)											
8	randomised trials	serious [a]	not serious[b]	not serious	serious [c]	publication bias strongly suspected [d]	676/1141 (59.2%)	663/1063 (62.4%)	RR 0.95 (0.90 to 1.01)	31 fewer per 1,000 (from 6 more to 62 fewer)	⊕◯◯◯ VERY LOW
Preventing Cold/ARI episodes (assessed with: all micronutrients (1 Zinc, 8 Vitamin D))											
9	randomised trials	serious [a,e]	not serious [b]	not serious	serious [f]	publication bias strongly suspected [g]	681/1161 (58.7%)	667/1080 (61.8%)	RR 0.96 (0.90 to 1.01)	25 fewer per 1,000 (from 6 more to 62 fewer)	⊕◯◯◯ VERY LOW
Duration of Cold/ARI episodes (assessed with: Zinc)											
6	randomised trials	serious [h]	serious [i]	not serious	not serious [j]	none [k]	214	207	-	MD 2.25 days lower (1.12 lower to 3.39 lower)	⊕⊕◯◯ LOW
Duration of Cold/ARI episodes (assessed with: Vitamin D)											
5	randomised trials	serious [l]	not serious [b]	not serious	serious [m]	none [n]	528	494	-	MD 0.14 days lower (0.48 lower to 0.2 higher)	⊕⊕◯◯ LOW
Duration of Cold/ARI episodes (assessed with: all micronutrients (5 Zinc, 3 Vitamin D))											

2

11	randomised trials	serious [o]	serious [p]	not serious	not serious [q]	none [k]	742	701	-	MD 1.36 days lower (0.29 lower to 2.43 lower)	⊕⊕◯◯ LOW

CI: Confidence interval; RR: Risk ratio; MD: Mean difference

Explanations:

a. Serious design limitations: All but 2 trials [4,7] had other bias or unclear other bias due to detection bias (insufficient statistical power [5,6,9], or no clinical/laboratory confirmation of URTI [6,8,9]) and recall bias [3]. Incomplete outcome data was also present in 1 trial [5] and unclear in another [3].

b. Low statistical heterogeneity: I^2 = 0%.

c. Sufficiently large sample size (N=2 204) but the 95% CI overlaps no effect.

d. Funnel plot indicated slight asymmetry. The suggested missing studies were observed to be broadly in the area of non-significance, indicating that publication bias is a plausible cause of funnel asymmetry. Studies also generally had small to moderate sample sizes.

e. Design limitation: Selection bias and detection bias is present and the trial has unclear reporting and attrition bias. But the trial has relatively smaller sample size compared to overall population.

f. Sufficiently large sample size (N=2 242) but the 95% CI overlaps no effect.

g. Funnel asymmetry observed on the right side of the plot, in the areas with low and mid statistical significance in a contoured funnel plot. Plausible that publication bias is the reason for asymmetry.

h. All but 2 trials had other bias due to insufficient statistical power [13], lack of clinical/laboratory confirmation of URTI [11,15], or presence of side intervention which could interfere with the observed effects [14]. 3 trials have serious design limitations, with unclear sequence generation and allocation concealment [15-17], out of which 2 have unclear group comparability [15,17] and 1 had significantly different groups at baseline [16]. Significantly different baseline characteristics which could affect outcome was also observed in another study [14].

i. Serious inconsistency: High statistical heterogeneity (I^2 =83%).

j. No serious imprecision: The total sample size (N = 421) is higher than the optimal information size (N = 197) needed to detect a one-day difference in cold duration (α = 0.05, 80% power) assuming a mean of 7 days (SD 6 days). However, the 95% CI (-3.39, -1.12) crossed the minimally important difference of one day but did not include the null effect.

k. Asymmetry was not detected in funnel plot. Contoured funnel plots showed asymmetry but most suggested missing studies lie in regions of high statistical significance, reducing plausibility that publication bias is the underlying cause of this funnel asymmetry.

l. Design limitations: Other bias due to insufficient statistical power and/or self-reported illness was unclear or present in all but 1 trials. Selective reporting was unclear in 2 trials [6,8], of which 1 trial also had unclear group comparability [8].

m. Total sample size = 1 022. The optimal information size to detect a one-day difference in cold duration (α = 0.05, 80% power) assuming a mean of 7 days (SD 8 days) was 503 participants. However, the 95% CI (-0.48, 0.20) included the null effect.

n. Slight asymmetry was detected in the funnel plot, which was in the area of low significance in a contoured funnel plot. However, the number of studies was too low to determine if publication bias could be the cause for plot asymmetry.

o. Design Limitations: All but 3 trials had other bias due to insufficient statistical power [13], lack of clinical/laboratory confirmation of URTI [8,10,11,15], or both [6,9]; or performance bias [14]. 5 trials have unclear [8,15,17] or significantly different group comparability [14,16] and 4 trials had unclear selective reporting [8,11,13,15].

p. Serious inconsistency: High statistical heterogeneity (I^2 =91%) which is significant (P<0.0001.)

q. No serious imprecision: The total sample size (N = 1 443) is higher than the optimal information size (N = 673) needed to detect a one-day difference in cold duration (α = 0.05, 80% power) assuming a mean of 7 days (SD 8 days). However, the 95% CI (-2.43, -0.29) crossed the minimally important difference of one day but did not include the null effect.

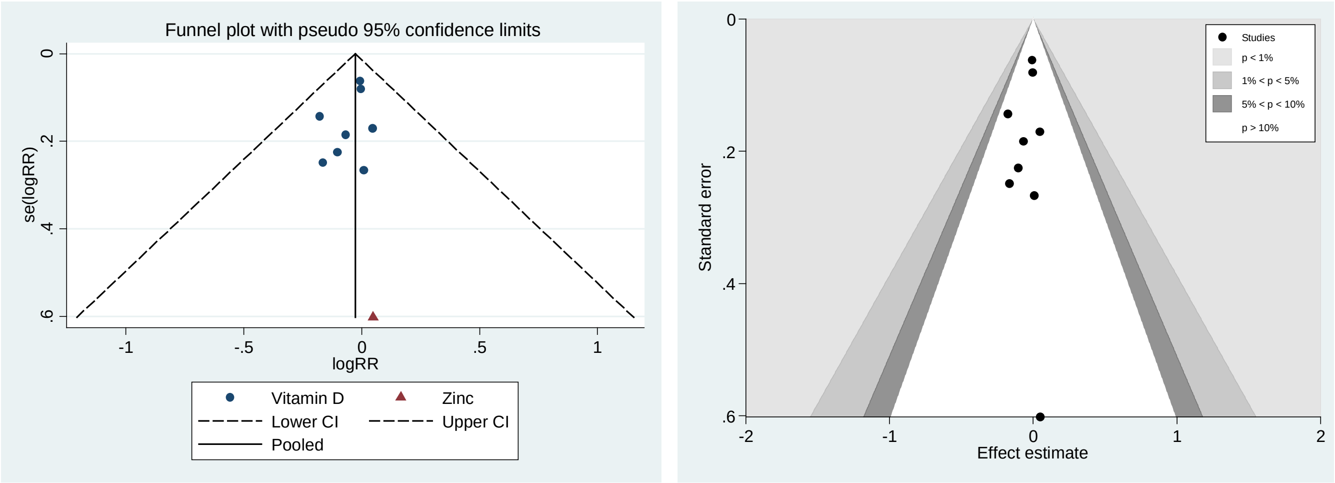

(a) (c)

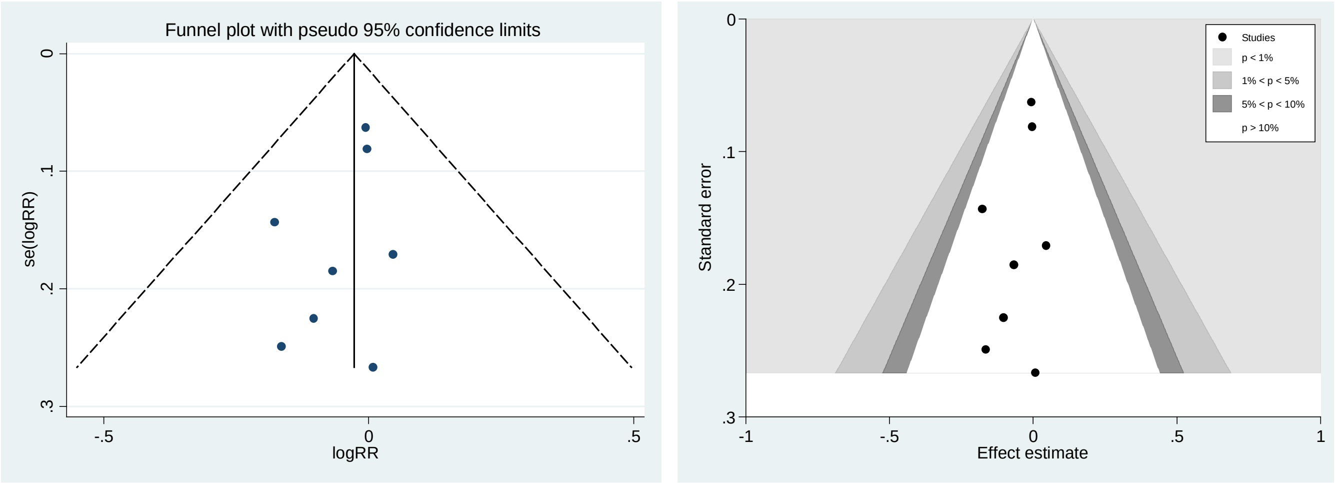

(b) (d)

Figure S1. Funnel plots for assessing the publication bias in studies reporting prevention of colds through micronutrient supplementation as an outcome: conventional funnel plots assessing the risk of publication bias in all studies supplementing (**a**) micronutrients or (**b**) vitamin D singly to prevent cold incidence; contoured funnel plots assessing the risk of publication bias in studies supplementing (**c**) micronutrients or (**d**) vitamin D singly to prevent cold incidence.

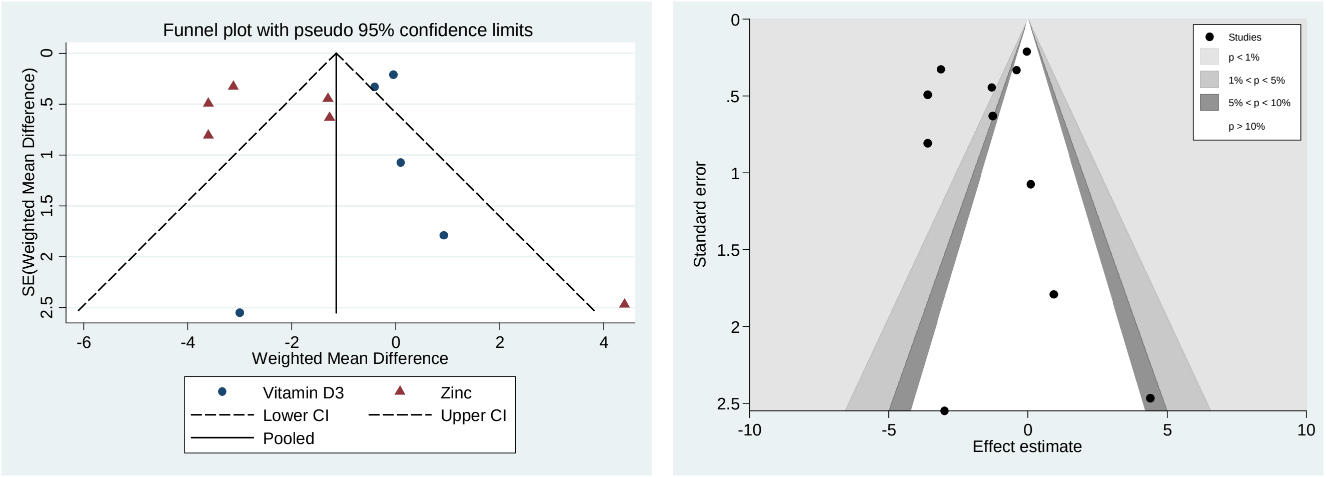

(a) (d)

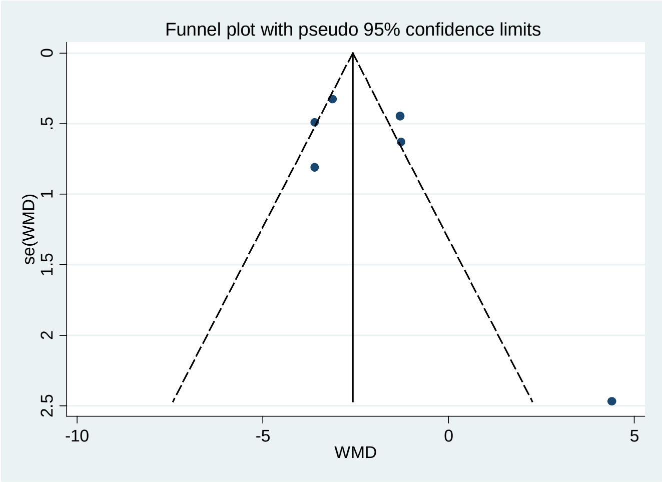

(**b**)

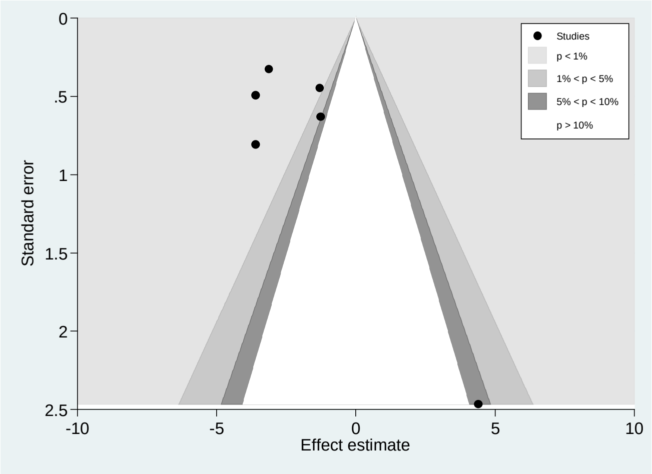

(**e**)

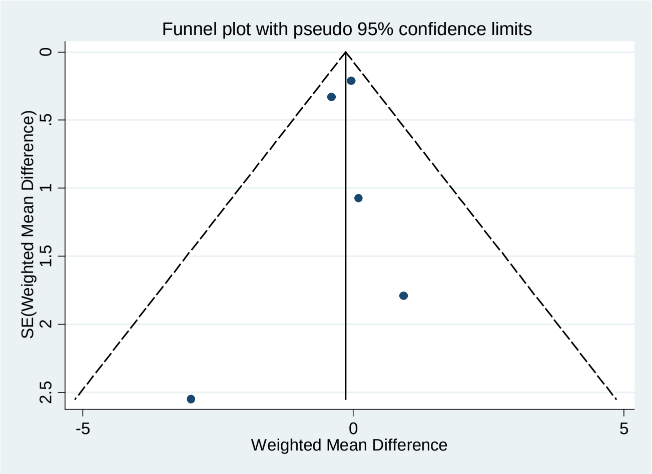

(**c**)

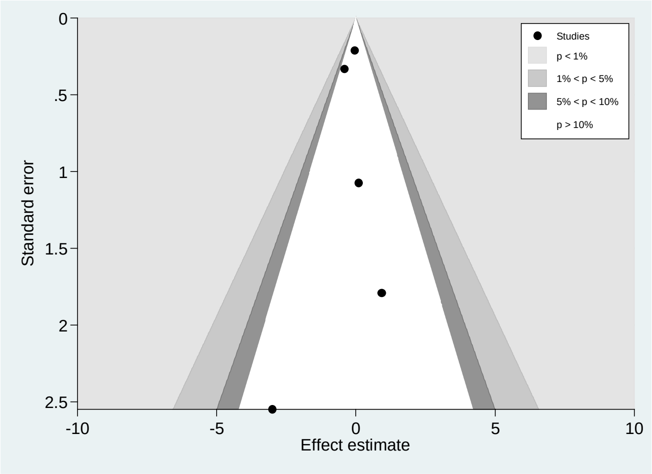

(**f**)

Figure S2. Funnel plots for assessing the publication bias in studies reporting management of cold duration through providing micronutrients as an outcome: conventional funnel plots assessing the risk of publication bias in all studies providing (**a)** micronutrients, (**b**) zinc or (**c**) vitamin D singly to shorten cold duration; contoured funnel plots assessing the risk of publication bias in studies providing (**d)** micronutrients, (**e**) zinc or (**f**) vitamin D singly to shorten cold duration.

References

1. Hemilä, H.; Kaprio, J.; Albanes, D.; Heinonen, O.P.; Virtamo, J. Vitamin C, vitamin E, and beta-carotene in relation to common cold incidence in male smokers. *Epidemiology* **2002**, *13*, 32-37, doi:10.1097/00001648-200201000-00006.
2. Hemilä, H.; Virtamo, J.; Albanes, D.; Kaprio, J. Vitamin E and Beta-Carotene Supplementation and Hospital-Treated Pneumonia Incidence in Male Smokers. *Chest* **2004**, *125*, 557-565, doi:10.1378/chest.125.2.557.
3. de Gruijl, F.R.; Pavel, S. The effects of a mid-winter 8-week course of sub-sunburn sunbed exposures on tanning, vitamin D status and colds. *Photochemical & photobiological sciences : Official journal of the European Photochemistry Association and the European Society for Photobiology* **2012**, *11*, 1848-1854, doi:10.1039/c2pp25179e.

4. Goodall, E.C.; Granados, A.C.; Luinstra, K.; Pullenayegum, E.; Coleman, B.L.; Loeb, M.; Smieja, M. Vitamin D3 and gargling for the prevention of upper respiratory tract infections: A randomized controlled trial. *BMC Infectious Diseases* **2014**, *14*, doi:10.1186/1471-2334-14-273.
5. Laaksi, I.; Ruohola, J.P.; Mattila, V.; Auvinen, A.; Ylikomi, T.; Pihlajamäki, H. Vitamin D supplementation for the prevention of acute respiratory tract infection: A randomized, double-blinded trial among young finnish men. *Journal of Infectious Diseases* **2010**, *202*, 809-814, doi:10.1086/654881.
6. Li-Ng, M.; Aloia, J.F.; Pollack, S.; Cunha, B.A.; Mikhail, M.; Yeh, J.; Berbari, N. A randomized controlled trial of vitamin D3 supplementation for the prevention of symptomatic upper respiratory tract infections. *Epidemiology and Infection* **2009**, *137*, 1396-1404, doi:10.1017/S0950268809002404.
7. Murdoch, D.R.; Slow, S.; Chambers, S.T.; Jennings, L.C.; Stewart, A.W.; Priest, P.C.; Florkowski, C.M.; Livesey, J.H.; Camargo, C.A.; Scragg, R. Effect of vitamin D3 supplementation on upper respiratory tract infections in healthy adults: the VIDARIS randomized controlled trial. *Jama* **2012**, *308*, 1333-1339, doi:10.1001/jama.2012.12505.
8. Rees, J.R.; Hendricks, K.; Barry, E.L.; Peacock, J.L.; Mott, L.A.; Sandler, R.S.; Bresalier, R.S.; Goodman, M.; Bostick, R.M.; Baron, J.A. Vitamin D3 supplementation and upper respiratory tract infections in a randomized, controlled trial. *Clinical Infectious Diseases* **2013**, *57*, 1384-1392, doi:10.1093/cid/cit549.
9. Shimizu, Y.; Ito, Y.; Yui, K.; Egawa, K.; Orimo, H. Intake of 25-hydroxyvitamin D3 reduces duration and severity of upper respiratory tract infection: A randomized, double-blind, placebo-controlled, parallel group comparison study. *Journal of Nutrition, Health and Aging* **2018**, *22*, 491-500, doi:10.1007/s12603-017-0952-x.
10. Simpson, S.; van der Mei, I.; Stewart, N.; Blizzard, L.; Tettey, P.; Taylor, B. Weekly cholecalciferol supplementation results in significant reductions in infection risk among the vitamin D deficient: results from the CIPRIS pilot RCT. *BMC Nutrition* **2015**, *1*, 7.
11. Douglas, R.M.; Miles, H.B.; Moore, B.W.; Ryan, P.; Pinnock, C.B. Failure of effervescent zinc acetate lozenges to alter the course of upper respiratory tract infections in Australian adults. *Antimicrobial agents and chemotherapy* **1987**, *31*, 1263-1265.
12. Eby, G.A.; Davis, D.; Halcomb, W. Reduction in duration of common colds by zinc gluconate lozenges in a double-blind study. *Antimicrobial agents and chemotherapy* **1984**, *25*, 20-24.
13. Godfrey, J.C.; Sloane, B.C.; Smith, D.S.; Turco, J.H.; Mercer, N.; Godfrey, N.J. Zinc Gluconate and the Common Cold: A Controlled Clinical Study. *Journal of International Medical Research* **1992**, *20*, 234-246, doi:10.1177/030006059202000305.

14. Mossad, S.B.; Macknin, M.L.; Medendorp, S.V.; Mason, P. Zinc Gluconate Lozenges for Treating the Common Cold: A Randomized, Double-Blind, Placebo-Controlled Study. *Annals of Internal Medicine* **1996**, *125*, 81-88, doi:10.7326/0003-4819-125-2-199607150-00001.
15. Petrus, E.J.; Lawson, K.A.; Bucci, L.R.; Blum, K. Randomized, double-masked, placebo-controlled clinical study of the effectiveness of zinc acetate lozenges on common cold symptoms in allergy- tested subjects. *Current Therapeutic Research - Clinical and Experimental* **1998**, *59*, 595-607, doi:10.1016/S0011-393X(98)85058-3.
16. Prasad, A.S.; Fitzgerald, J.T.; Bao, B.; Beck, F.W.; Chandrasekar, P.H. Duration of symptoms and plasma cytokine levels in patients with the common cold treated with zinc acetate: a randomized, double-blind, placebo-controlled trial. *Annals of Internal Medicine* **2000**, *133*, 245-252.
17. Prasad, A.S.; Beck, F.W.; Bao, B.; Snell, D.; Fitzgerald, J.T. Duration and severity of symptoms and levels of plasma interleukin-1 receptor antagonist, soluble tumor necrosis factor receptor, and adhesion molecules in patients with common cold treated with zinc acetate. *The Journal of infectious diseases* **2008**, *197*, 795-802, doi:10.1086/528803.
18. Turner, R.B.; Cetnarowski, W.E. Effect of treatment with zinc gluconate or zinc acetate on experimental and natural colds. *Clinical Infectious Diseases* **2000**, *31*, 1202-1208, doi:10.1086/317437.
19. Veverka, D.V.; Wilson, C.; Martinez, M.A.; Wenger, R.; Tamosuinas, A. Use of zinc supplements to reduce upper respiratory infections in United States Air Force Academy cadets. *Complementary therapies in clinical practice* **2009**, *15*, 91-95, doi:10.1016/j.ctcp.2009.02.006.
20. Weismann, K.; Jakobsen, J.P.; Weismann, J.E.; Hammer, U.M.; Nyholm, S.M.; Hansen, B.; Lomholt, K.; Schmidt, K. Zinc gluconate lozenges for common cold. *Dan Med Bull* **1990**, *37*, 279-281.
